# Metabolic engineering of microorganisms for production of aromatic compounds

**DOI:** 10.1186/s12934-019-1090-4

**Published:** 2019-02-26

**Authors:** Damla Huccetogullari, Zi Wei Luo, Sang Yup Lee

**Affiliations:** 1 Metabolic and Biomolecular Engineering National Research Laboratory, Department of Chemical and Biomolecular Engineering (BK21 Plus Program) and Institute for the BioCentury, Korea Advanced Institute of Science and Technology (KAIST), Daejeon, 34141 Republic of Korea; 2 Systems Metabolic Engineering and Systems Healthcare Cross-Generation Collaborative Laboratory, KAIST, Daejeon, 34141 Republic of Korea; 3 BioProcess Engineering Research Center and Bioinformatics Research Center, KAIST, Daejeon, 34141 Republic of Korea

**Keywords:** Aromatic compounds, Metabolic engineering, Synthetic biology, Shikimate pathway, Phenylalanine, Tyrosine, Tryptophan

## Abstract

Metabolic engineering has been enabling development of high performance microbial strains for the efficient production of natural and non-natural compounds from renewable non-food biomass. Even though microbial production of various chemicals has successfully been conducted and commercialized, there are still numerous chemicals and materials that await their efficient bio-based production. Aromatic chemicals, which are typically derived from benzene, toluene and xylene in petroleum industry, have been used in large amounts in various industries. Over the last three decades, many metabolically engineered microorganisms have been developed for the bio-based production of aromatic chemicals, many of which are derived from aromatic amino acid pathways. This review highlights the latest metabolic engineering strategies and tools applied to the biosynthesis of aromatic chemicals, many derived from shikimate and aromatic amino acids, including l-phenylalanine, l-tyrosine and l-tryptophan. It is expected that more and more engineered microorganisms capable of efficiently producing aromatic chemicals will be developed toward their industrial-scale production from renewable biomass.

## Background

Petroleum-derived chemicals have been essential in modern society because of their high demand in manufacturing fuels, solvents and materials among others. To cope with the grand concerns associated with global climate change and limited availability of fossil resources in the future, there has been much effort exerted to develop microbial strains capable of producing diverse chemicals and materials from renewable resources. Aromatic chemicals are important in chemical, food, polymer and pharmaceutical industries since they serve various purposes [1, 2]. While bio-based processes for the production of a few aromatics have been commercialized [3], the majority of aromatic compounds are chemically synthesized due to the inefficiency of their biological production or even the lack of an appropriate bioproduction process. With the rapid advances in systems metabolic engineering tools and strategies, however, microbial production of aromatic compounds has seen a considerable progress over the past few years [4]. By this approach, chemical processes for the synthesis of various aromatics using petroleum-derived benzene, toluene and xylene (BTX) as the starting materials can be replaced by bio-based sustainable and environmentally friendly processes using renewable non-food resources.

**Fig. 1:**
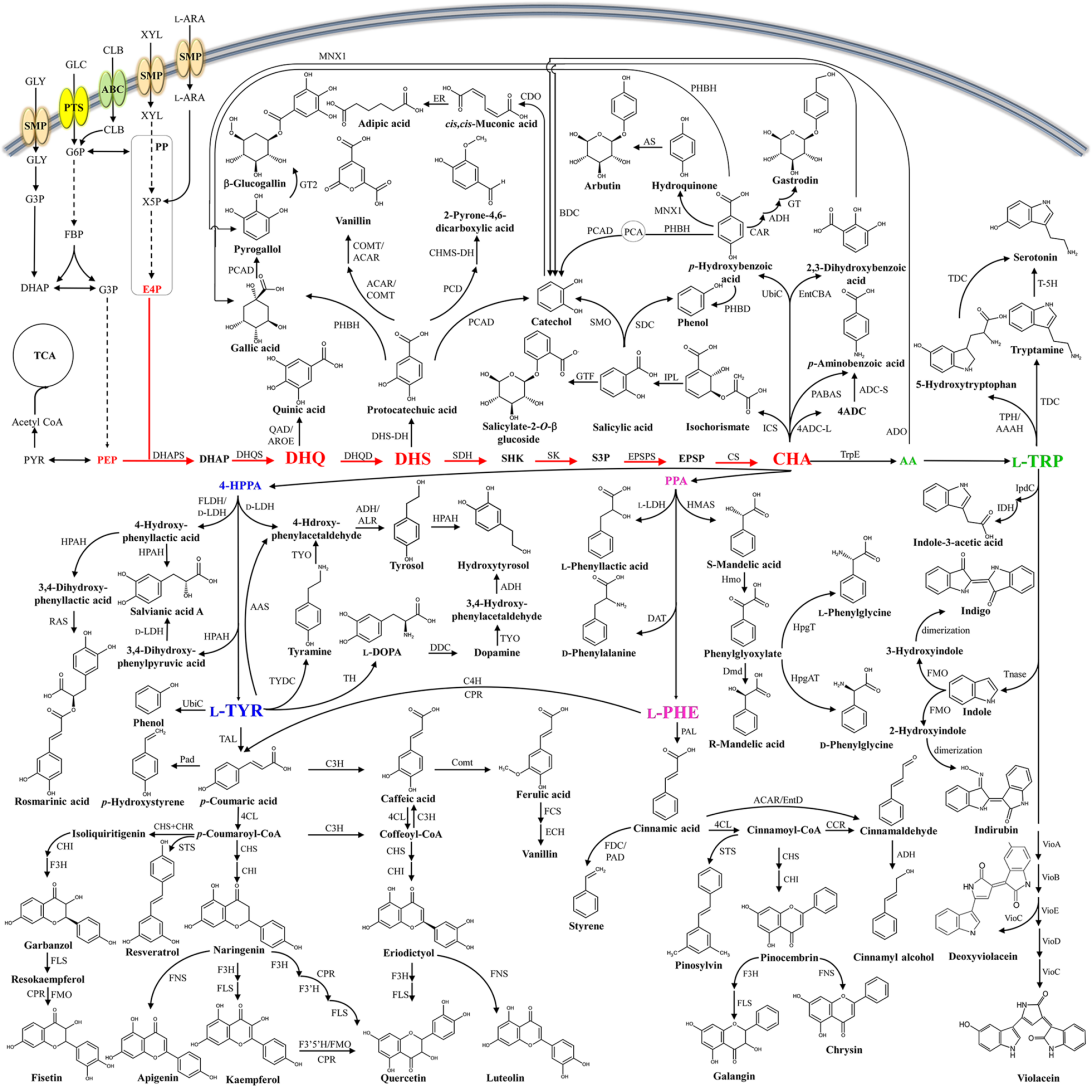
De novo biosynthesis of various aromatic compounds derived from the SHK and aromatic amino acid biosynthesis pathway. Abbreviations for metabolites: 4-HPPA: 4-hydroxyphenylpyruvate; AA: anthranilic acid; ABC: ATP-binding cassette transporter; l-ARA: l-arabinose; CHA: chorismate; CLB: cellobiose; DAHP: 3-deoxy-d-arabinoheptulosonate 7-phosphate; DHAP: dihydroxyacetone phosphate; DHQ: 3-dehydroquianate; DHS: 3-dehydroshikimate; E4P: erythrose 4-phosphate; EPSP: 5-enolpyruvyl-shikimate 3-phosphate; FBP: fructose 1,6-biphosphate; G3P: glyceraldehyde 3-phosphate; G6P: glucose 6-phosphate; GLC: glucose; GLY: glycerol; PCA: protocatechuic acid; PEP: phosphoenolpyruvate; l-PHE: l-phenylalanine; PP: pentose phosphate; PPA: phenylpyruvate; PTS: phosphotransferase system; PYR: pyruvate; S3P: shikimate-3-phosphate; SHK: shikimate; SMP: proton symporter; TCA: tricarboxylic acid; l-TRP: l-tryptophan; l-TYR: l-tyrosine; X5P: xylose 5-phosphate; XYL: xylose. Abbreviations for enzymes: 4ADCL: 4-amino-4-deoxychorismate lyase; 4-CL: 4-coumarate:CoA ligase; AAAH: aromatic amino acid hydroxylase; AAS: aromatic acetaldehyde synthase; AAT: aromatic amino acid transaminase; ACAR: aromatic carboxylic acid reductase; ADCS: aminodeoxychorismate synthase; ADH: alcohol dehydrogenase; AntABC: anthranilate 1,2-dioxygenase; AROE: shikimate dehydrogenase; AS: hydroquinone glucosyl transferase; BDC: 2,3-DHBA decarboxylase; C3H: *p*-coumarate 3-hydroxylase; C4H: cinnamic acid decarboxylase; CAR: carboxylic acid reductase; CCR: cinnamoyl-CoA reductase; CDO: catechol 1,2-dioxygenase; CHI: chalcone isomerase; CHS: chalcone synthase; Comt: caffeate *O*-methyltransferase; COMT: catechol-*O*-methyltransferase; CPR: cytochrome P450 reductase; CS: chorismate synthase; DAT: d-amino acid transferase; DBR: double bond reductase; DDC: l-DOPA decarboxylase; DHAPS: 3-deoxy-d-arabinoheptulosonate 7-phosphate synthase; DHQD: 3-dehydroquianate dehydratase; DHQS: 3-dehydroquianate synthase; DHS-DH: 3-dehydroshikimate dehydratase; Dmd: d-mandelate dehydrogenase; ECH: feruloyl-CoA hydratase/lyase; EntA: 2,3-dihydro-2,3-dihydroxybenzoate dehydrogenase; EntB: isochorismatase; EntC: isochorismate synthase; EntD: phosphopantetheinyl transferase; EPSPS: 5-enolpyruvyl-shikimate 3-phosphate synthase; ER: enoate reductase; F3H: flavanone 3-hydroxylase; F3′H: flavonoid 3-hydroxylase; F3′5′H: *F3′5′H*-encoded 3′,5′-hydroxylase; FCS: feruloyl-CoA synthetase; FDC: ferulate decarboxylase; FLDH: d-phenyllactate dehydrogenase; FLS: flavanol synthase; FMO: flavin-containing monooxygenase; FNS: flavone synthase; GT2: gallic acid glucosyltransferase; GTF: glucosyltransferase; HA-DH: hydroxyacyl-dehydrogenase; HmaS: l-4-hydroxymandelate synthase; Hmo: l-4-hydroxymandelate oxidase; HPAH: 4-hydroxyphenylacetate 3-hydroxylase; HpgAT: l-4-(hydroxyl)-phenylglycine aminotransferase; HpgT: l-4-hydroxyphenylglycine aminotransferase; ICS: isochorismate synthase; IDH: indole acetic acid dehydrogenase; IpdC: indole-3-pyruvic acid decarboxylase; IPL: isochorismate pyruvate lyase; d-LDH: d-lactate dehydrogenase; l-LDH: l-lactate dehydrogenase; MNX1: 4-hydroxybenzoate 1-hydroxylase; PABAS: *p*-aminobenzoate synthase; Pad: phenolic acid decarboxylase; PAD: phenylacrylate decarboxylase; PAL: phenylalanine ammonia lyase; PCD: protocatechuic acid 4,5-dioxygenase; PHBD: 4-hydroxybenzoate decarboxylase; PHBH: *p*-hydroxybenzoate hydroxylase; QAD: quinate dehydrogenase; RAS: rosmarinic acid synthase; SDC: salicylate decarboxylase; SK: shikimate kinase; SMO: salicylate monooxygenase; STS: stilbene synthase; T-5H: tryptamine 5-hydroxylase; TAL: tyrosine ammonia-lyase; TDC: tryptophan decarboxylase; Tnase: tryptophanase; TPH: tryptophan 5-hydroxylase; TPL: tyrosine phenol-lyase; TrpE: anthranilate synthase; TYDC: tyrosine decarboxylase; TYO: tyramine oxidase; UbiC: chorismate lyase; VioA: l-tryptophan oxidase; VioB: iminophenyl-pyruvate dimer synthase; VioC: violacein synthase; VioD: protoviolaceinate synthase; VioE: violacein biosynthesis protein. Continuous arrows show single enzymatic reactions, and dashed arrows show multiple enzymatic reactions

The chemicals that have been produced by microorganisms can be classified as natural (native or non-native) and non-natural ones [5, 6]. Among aromatic compounds produced in microbial systems, for example, the plant-originated aromatics such as phenolic acids, flavonoids, stilbenoids, coumarins and their derivatives are natural chemicals but non-native to many microorganisms [7]. On the contrary, some other common aromatic chemicals including *cis*,*cis*-muconic acid and styrene are non-natural chemicals. Since the vast majority of microbially produced aromatic chemicals are derived from shikimate (SHK) and aromatic amino acids including l-phenylalanine (l-PHE), l-tyrosine (l-TYR) and l-tryptophan (l-TRP), we herein categorize these aromatic compounds into (i) intermediates and derivatives of the SHK pathway, and (ii) aromatic amino acids (l-PHE, l-TYR and l-TRP) and their derivatives, on the basis of different biosynthetic pathway modules from which they are derived (Fig. 1).

In this paper, we review the current status of microbial production of various aromatic compounds focusing on metabolic engineering tools and strategies employed. In particular, those aromatic compounds that have been produced in engineered microbial cell factories from renewable feedstocks are described according to the different biosynthetic modules, with highlights on the construction of biosynthesis pathways, enzyme engineering, modulation of metabolic fluxes, omics- and modeling-based technologies. These strategies and design principles can also be implemented in the future for microbial production of new and more complex aromatic compounds.

## Engineering the central carbon metabolism for aromatic precursors

Metabolic intermediates in the SHK pathway and in the aromatic amino acid biosynthesis pathway are good precursors for the production of various aromatic compounds. However, these metabolites do not typically accumulate during the metabolic reactions due to the tight regulation. To enhance metabolic fluxes towards the SHK and aromatic amino acid pathways, several well-defined conventional strategies that have been employed include: (i) replacing the native phosphotransferase system (PTS)-mediated glucose uptake system with alternatives, (ii) increasing availability of the two key SHK pathway precursors, phosphoenolpyruvate (PEP) and erythrose-4-phospahte (E4P), (iii) down-regulating carbon fluxes towards competing and catabolic pathways of precursors, and (iv) enhancing the bottleneck enzyme reactions.

Phosphoenolpyruvate is considered a rate-controlling precursor in the SHK pathway since it is also needed for the PTS system responsible for the uptake and phosphorylation of particular sugars and sugar derivatives and also for the biosynthesis of cellular materials [8]. To decrease PEP consumption by PTS during sugar uptake, alternative carbon sources such as xylose and l-arabinose that do not require PTS for their uptake have been employed. It was reported that the use of xylose as a sole carbon source resulted in production of 820.18 mg/L of 4-hydroxymandellic acid (4-HMA) in an engineered *Escherichia coli*, whereas the use of glucose yielded 747.13 mg/L 4-HMA [9]. Also, the use of a non-PTS system which does not consume PEP is an alternative way for sugar transport. For example, through the facilitated diffusion with ATP-based phosphorylation, glucose can be first transported by galactose permease (GalP), and then phosphorylated by glucokinase in *E. coli* [10]. In a mutant *E. coli* strain lacking PTS, the carbon flux was successfully increased towards aromatic amino acid pathway due to the elevated glucokinase activity leading to rapid glucose consumption, although the specific cell growth rate was lower than that of the wild-type strain [11]. To investigate the effect of non-PTS system on production of 3-deoxy-d-arabinoheptulosonate 7-phosphate (DAHP) as a starting precursor in the SHK pathway, the performance of 3-dehydroquinate (DHQ) synthase-deficient *E. coli* strains having PTS and non-PTS were compared. In the *E. coli* strain having the non-PTS system, the yield of DAHP on glucose was 1.65-fold higher than that obtained with the *E. coli* strain having the PTS system [12]. Moreover, the use of a non-PTS system together with overexpression of several key genes that encode DAHP synthase, transketolase and chorismate (CHA) mutase-prephenate dehydratase, resulted in a l-PHE overproducer strain of *E. coli* with the yield of 0.33 g/g glucose [13]. The non-PTS system of *Zymomonas mobilis* was also used to improve the cellular PEP availability in combination with the native non-PTS system in *E. coli*. In particular, the glucose facilitator encoded by *glf* in *Z. mobilis* transports glucose by facilitated diffusion, whereas that encoded by *galP* in *E. coli* transports glucose by proton symport system. Glucose transport by both systems is followed by the glucokinases using ATP-based phosphorylation. When compared to the native non-PTS in *E. coli*, the heterologous *Z. mobilis* non-PTS system was more efficient since it enabled production of 60 g/L DHS with higher productivity [14]. Besides the well-known non-PTS systems, a new non-PTS system was recently reported as myo-inositol transporter (encoded by *iolT1*) in *Corynebacterim glutamicum* [15]. Through deletion of the transcriptional regulator protein encoded by *iolR* that inhibits the expression of *iol* gene cluster, the non-PTS system of *C. glutamicum* was activated, leading to production of 4.01 g/L of *cis*,*cis*-muconic acid with the highest yield of 22% mol/mol glucose in *E. coli* [16].

To increase the PEP pool, several other useful strategies employed include: overexpression of PEP-forming enzymes (i.e., PEP synthase and PEP carboxykinase) or inactivation of PEP-degrading enzymes (i.e., pyruvate kinases and PEP carboxylase) [3]. Although the inactivation of PEP-degrading enzymes modulated carbon flux towards the SHK pathway, cell growth decreased by almost a half probably due to the accumulation of byproducts such as acetate and pyruvate and decrease of TCA intermediates. On the other hand, the increase in the PEP synthase activity positively affected aromatic compound production, especially when transketolase was also overexpressed [17].

Increase of the E4P pool also improves formation of DAHP. Since the first studies that showed the contribution of E4P to the synthesis of DAHP [18] and aromatic compounds [19], its overexpression has been one of the strategies popularly used in aromatic compounds production. As an intermediate of the pentose phosphate (PP) pathway, E4P is synthesized from sedo-heptulose-1,7-biphosphate [20].

## Engineering the SHK pathway and its derivatives

The SHK pathway links the central carbon metabolism to the biosynthesis of aromatic amino acids including l-TRP, l-TYR and l-PHE [21]. This pathway comprising seven successive enzymatic reactions leads to the biosynthesis of CHA, a key aromatic precursor, which is also a branch point for the biosynthesis of three aromatic amino acids as well as diverse aromatic compounds [22, 23]. Intermediates in the SHK pathway are also precursors for the biosynthesis of diverse secondary metabolites especially in plants [24]. As these intermediates are important for the biosynthesis of various derivative aromatic compounds, development of strategies for their efficient production is a key for the de novo synthesis of numerous aromatic compounds.

Since the first chemical synthesis of *p*-hydroxybenzoic acid (PHBA) from benzene on industrial scale in 1980’s, PHBA and its derivatives have been extensively used for the synthesis of liquid crystal, paraben (a preservative used in cosmetic and pharmaceutical products), phenol and others [25, 26]. Alternatively, PHBA is naturally produced through one-step conversion from CHA by the *ubiC*-encoded CHA pyruvate-lyase during the synthesis of ubiquinone in *E. coli*, or through cascade reactions from CHA through phenylpropanoids, i.e., *p*-coumaric acid, in the plant secondary metabolism [25, 27]. By mimicking this natural pathway of *E. coli*, metabolically engineered *C. glutamicum* was able to produce PHBA to the highest titer and yield of 36.6 g/L and 41% (mol/mol), respectively [26], which were higher than those achieved with engineered *E. coli* [25]. Apart from the conventional metabolic engineering strategies such as the elimination of competing pathways through inactivation of enzymes involved in the central carbon metabolism (i.e., *ldhA*-encoded lactate dehydrogenase, *pyk*-encoded pyruvate kinase, *hdpA*-encoded haloacid dehalogenase) and in SHK pathway (*qsuB*-encoded dehydroshikimate dehydratase, *qsuD*-encoded shikimate dehydrogenase, *pobA*-encoded 4-hydroxybenzoate hydroxylase), the use of PHBA-resistant UbiC from *Providencia rustigianii* and growth-arrested bioprocess proved to be effective for increasing PHBA production in the engineered *C. glutamicum*. Additionally, a mutated *aroG* gene from *E. coli* and the native *aroCKB* that encodes CHA synthase, SHK kinase and DHQ synthase, respectively, were overexpressed via chromosomal integration [26]. In *E. coli*, the enhanced central carbon flux towards CHA was accomplished by overexpressing transketolase in PP pathway and feedback insensitive DAHP synthase (AroF^fbr^), DHQ synthase, SHK kinase, 5-enolpyruvylshikimate 3-phosphate (EPSP) synthase and CHA synthase in SHK pathway [25]. Although *Saccharomyces cerevisiae* can tolerate high concentration of PHBA up to 38.3 g/L [28], the engineered yeast produced PHBA only to milligram levels under either batch operation in shake-flask or pulsed-feeding in fermenter mainly due to excessive by-product formation [28, 29]. An *E. coli*–*E. coli* co-culture system has also been developed for PHBA production. The *E. coli* strain harboring the upstream PHBA pathway was engineered to produce and secrete DHS, which was assimilated by the engineered *E. coli* strain harboring the downstream pathway through the DHS importer ShiA to produce PHBA. Also, the sugar utilization pathways in both strains were manipulated for independent carbon source utilization. When these two *E. coli* strains were co-cultured, 2.3 g/L of PHBA was produced from a mixture of glucose and xylose [30].

*cis*,*cis*-Muconic acid is a dicarboxylic acid used as a precursor in the chemical synthesis of adipic acid, a major building block for the synthesis of nylon. Currently, *cis*,*cis*-muconic acid is produced by petrochemical processes. Biological production of *cis*,*cis*-muconic acid has also been developed, either through biotransformation of aromatic substrates (e.g., benzoate) or via de novo biosynthesis from renewable sources (e.g., lignin and glucose) [31]. On the basis of its presence as an intermediate metabolite both in the β-ketoadipate pathway during aromatic hydrocarbon degradation in bacteria [32–34] and the ubiquinone biosynthesis pathway [35], *cis*,*cis*-muconic acid has been biosynthesized through two different pathways using DHS or CHA as a SHK pathway precursor [36]. After the first report on microbial production of *cis*,*cis*-muconic acid employing DHS as precursor [37], the strategies have been mainly focused on optimizing the heterologous pathway comprising DHS dehydratase, PCA decarboxylase and catechol 1,2-dioxygenase. The highest titer of *cis*,*cis*-muconic acid reported is 36.8 g/L using an engineered *E. coli* harboring the *aroZ*-encoded DHS dehydratase and *aroY*-encoded PCA decarboxylase from *Klebsiella pneumonia*, and *catA*-encoded catechol 1,2-dioxygenase from *Acinetobacter calcoacetius* [38]. By using the same metabolic engineering strategy with DHS as precursor, 2-pyrone-4,6-dicarboxylic acid has also been produced. The highest 2-pyrone-4,6-dicarboxylic acid titer of 16.7 g/L was achieved by combining the downstream heterologous bioconversion steps catalyzed by *asbF*-encoded DHS dehydratase from *Bacillus thuringiensis*, *pmdAB*-encoded PCA 4,5-dioxygenase and *pmdC*-encoded 4-carboxy-2-hydroxymuconate-6-semialdehyde dehydrogenase from *Comamonas testosteroni* [39]. Using an *E. coli*–*E. coli* co-culture system, 4.7 g/L of *cis*,*cis*-muconic acid was produced from a glucose/xylose mixture [30].

While earlier studies mainly focused on producing *cis*,*cis*-muconic acid from DHS, the recent studies employed CHA derivatives as precursors, i.e., anthranilic acid, 2,3-dihydroxybenzoic acid (2,3-DHBA), PHBA, salicylic acid [40–43]. Among them, the pathway derived from salicylic acid yielded gram-level of *cis*,*cis*-muconic acid in shake-flask experiments [43], whereas the production levels by using the other precursors remained quite low. For instance, the biosynthetic pathway of *cis*,*cis*-muconic acid derived from anthranilic acid was inspired by the anthranilate degradation pathway followed by subsequent formation of catechol and *cis*,*cis*-muconic acid in *Actinobacter* and *Pseudomonas* [40]. The *Rhizobium radiobacter* 2,3-DHBA decarboxylase (BDC) was also reported to convert 2,3-DHBA to catechol through its promiscuous activity [41]. Additionally, *S. cerevisiae* has also been engineered for biosynthesis of *cis*,*cis*-muconic acid from DHS by introducing candidate genes from different organisms, but *cis*,*cis*-muconic acid production was rather inefficient (Table 1) [28, 44, 45]. When comparing the recent microbial platforms for *cis*,*cis*-muconic acid production, one remarkable example is the engineered *Pseudomonas putida* strain that showed enhanced activity of *aroY*-encoded PCA decarboxylase from *Enterobacter cloacae*, resulting from co-expression with other small proteins called EcdB and EcdD. These proteins led to the conversion of PCA to catechol rather than the intermediate in β-ketoadipate pathway [46]. By combining synthetic *cis*,*cis*-muconic acid pathway with further bioconversion reaction catalyzed by enoate reductase (ER) from *Bacillus coagulans*, microbial production of adipic acid was achieved in engineered *S. cerevisiae*, although the final titer of adipic acid was low [47].

**Table 1:** The systems metabolic engineering strategies on shikimate pathway for the microbial production of aromatic compounds

Host	Product	Precursor	Carbon source	Titer	Time (h)	Bioprocess strategy	Systems metabolic engineering strategies	References
*E. coli*	PHBA	CHR	Glucose	12 g/L	72	Fed-batch (fermenter)	Overexpressing *ubiC* and particular genes in PP and SHK pathway	[25]
	*cis*, *cis*-Muconic acid	DHS	Glucose	36.8 g/L	48	Fed-batch (fermenter, 2 L)	Inserting three heterologous genes from different microorganisms, being responsible for the product synthesis, deleting SDH-coding gene	[38]
		AA	Glycerol/glucose	0.389 g/L	32	Batch (shake flask)	Optimizing AA pathway through overexpressing genes in PP and SHK pathway and shunting tryptophan biosynthesis; screening target genes being responsible for the biosynthesis from AA	[40]
		2,3-DHBA	Glycerol/glucose	0.480 g/L	48	Batch (shake flask)	Screening and characterization of new BDC enzyme from different organisms; overexpressing target genes; deleting gene responsible for degradation of 2,3-DHBA	[41]
		4HBA	Glucose	0.170 g/L	24	Batch (shake flask)	Integrating heterologous genes for the synthesis of CMA from precursor 4HBA to target product; over- and co-expressing native target genes and mutated ones; deleting genes in PEP–PTS	[42]
		SA	Glycerol/glucose	1.5 g/L	48	Batch (shake flask)	Shunting SHK pathway from chorismate to aromatic amino acids; overexpressing genes in CCM and SHK pathway; Integrating heterologous genes being responsible in SA anabolism and catabolism	[43]
	Salicylic acid	CHA	Glycerol/glucose	1.2 g/L	48	Batch (shake flask)		
		CHA	Glucose	11.5 g/L	48	Batch (fermenter, 2 L)	Replacing endogenous PTS system; Deleting genes of enzyme converting PEP to PYR; Integrating genes catalyzing product formation via CHR	[64]
	PABA	CHA	Glucose	4.8 g/L	48	Fed-batch (shake flask)	Overexpressing feedback resistance gene at entrance of SHK pathway and heterologous genes; the integration of fused type ADC synthase	[70]
	Gallic acid	DHS	Glucose	20 g/L	48	Fed-batch (fermenter)	Discovering side activity of *pobA* mutant to produce gallic acid from protocatechuic acid.	[73]
	Pyrogallol	2,3-DHBA	2,3-DHBA	0.893 g/L	24	Fed-batch (shake-flask)	Identifying and characterizing 2,3-DHBA MNX1 from different sources; Integrating *nahG*-encoded MNX1; Optimizing CCM and SHK pathway with EntCBA activity	[74]
				1.04 g/L			Autoxidation with oxygen scavenging agent ascorbic acid into the medium	
	Gallic acid	PHBA	Glucose/glycerol	1.266 g/L	36	Batch (shake-flask)	Mutating *pobA* (Y385F/T294A) from *Pseudomonas aeruginosa;* step-wise upstream pathway engineering with overexpression of *UbiC* to increase carbon flux to precursor	[75]
	Quinic acid	DHQ	Glucose	4.8 g/L	N.A	Batch (shake-flask)	Integration of gene encoding quinic acid dehydrogenase in *Klebsiella pneumaniae*	[76]
			Glucose	49 g/L	48	Fed-batch (fermenter, 2 L)	Integration of *aroE* encoded shikimate dehydrogenase	[77]
	PDC	DHS	Glucose	16.72 g/L	60	Fed-batch (fermenter, 6.6 L)	Overexpressing feedback resistant variant *aroG*, *ppsA* and *shiA*; Integrating three heterologous genes	[39]
*C. glutamicum*	PHBA	CHA	Glucose	36.6 g/L	24	Growth arrested bioprocess (fermenter, 1 L)	Deleting target genes in competing pathway in CCM and SHK pathway; Screening and introducing best *ubiC*-encoded enzyme; Stepwise overexpressing the genes in PP and SHK pathway	[26]
	PABA	CHA	Glucose	43 g/L	48	Fed-batch (fermenter, 1 L)	Screening *pab* genes from different microorganisms for target synthesis; Introducing fused ADC synthase from *C. callunae* and ADC4 lyase from *X. bovienii*	[71]
*P. putida*	*cis*, *cis*-Muconic acid	DHS	Glucose	4.92 g/L	54	Fed-batch (fermenter, 0.5 L)	Co-expression of two-genetically associated protein with PCA decarboxylase	[46]
*S. cerevisiae*	PHBA	CHA	Glucose	0.089 g/L	> 150	Pulse-feeding (fermenter, 1 L)	Deleting target genes for alleviated negative feedback; integration and overexpression of chorismate lyase originated from *E. coli*	[28]
			Glucose	0.148 g/L	N.A	Batch (shake flask)	Quorum sensing linked RNA interference for separate growth and production phase	[29]
	*cis*, *cis*-Muconic acid	DHS	Glucose	1.56 mg/L	170	Batch (shake flask)	Screening and combining best candidates of heterologous genes s catalyzing the reactions from DHS to cis–cis muconic acid	[44]
			Glucose	141 mg/L	108	Batch (shake flask)	Screening and combining target genes and their putative concerned with targeted product; overexpressing PP pathway specific gene and feedback resistant mutant gene in SHK pathway	[45]
	PABA	CHA	Glucose	0.034 g/L	> 150	Pulse-feeding (fermenter, 1 L)	Eliminating the genes on the entry towards aromatic amino acid pathway; overproducing gene related to product	[28]
			Glucose	> 0.068 g/L	130	Batch (shake flask)	Screening additional allels of target homologous 2 genes being responsible for PABA production; combining them each other for their overexpression	[69]
			Glycerol	0.215 g/L		Fed-batch (fermenter)		

Vanillin (4-hydroxy-3-methoxybenzaldehyde), a widely used fragrance and aroma agent in various industries, is one of the first aromatic compounds produced by engineered microbial systems. Its microbial production is considered as an eco-friendly alternative to the traditional methods (i.e., extraction from seedpods of orchids and lignin/hydrocarbon (guaiacol)-based synthesis) as reviewed elsewhere [48]. While vanillin is intrinsically synthesized as a secondary metabolite in *Vanilla planifolia* through pathways not fully understood [49], a few actinomycetes, such as *Streptomyces setonii* 75vi2 (recently known as *Amycolatopsis* sp. strain ATCC 39116) and *Amycolatopsis* sp. HR167, are also capable of producing vanillin as an intermediate in their ferulic acid degradation pathway [50, 51]. This degradation pathway can vary with initial reactions in different microorganisms, which has been previously reviewed in detail [49]. In addition, *Pseudomonas* sp. strain HR199 was also reported to synthesize vanillin from ferulic acid and eugenol by the contribution of native enzymes such as *fcs*-encoded feruloyl-CoA synthetase, *ech*-encoded enoyl-CoA hydratase/aldolase and *aat*-encoded B-ketothiolase [52]. This pathway is based on the so-called CoA-dependent retro-aldol mechanism, and it is inherently present in a number of microorganisms, i.e., *Delftia acidovorans* [53], *Pseudomonas fluorescens* AN103, *Amycolaptosis* sp. strain HR167 [49] and *P. putida* [54]. Metabolic engineering of a native vanillin producer *P. fluorescens* led to relatively high-level production of vanillin from ferulic acid [55]. On the other hand, a recombinant strain of *E. coli* XL1-Blue harboring the corresponding heterologous genes *fcs* and *ech* was also developed for vanillin production from ferulic acid. The vanillin titer was further improved to 5.14 g/L in shake-flask experiments by reducing the acetyl-CoA consumption through overexpression of *gltA* (encoding citrate synthase) and deletion of *icdA* (encoding isocitrate dehydrogenase) [56]. In another report, 7.8 g/L of vanillin was produced from ferulic acid by a two-stage process using *E. coli* whole-cell biocatalysts harboring coenzyme-independent decarboxylase/oxygenase [57]. Despite high titers of vanillin, the requirement of the expensive substrate ferulic acid is a drawback associated with the aforementioned biotransformation processes. To replace ferulic acid with a relatively inexpensive substrate, such as eugenol, an engineered *E. coli* was developed by introducing *vaoA*-encoded vanilly alcohol oxidase from *Penicillium simplicissimum*. This strain was able to produce 0.3 g/L of vanillin through two-step biotransformation process [58]. In addition to biotransforming ferulic acid, eugenol and isoeugenol for vanillin production, a number of studies have shown de novo vanillin production from renewable and even cheaper substrates such as glucose. In an initial study, an engineered *E. coli* strain expressing the *aroZ*-encoded DHS dehydratase from the mold fungus *Podospora anserine* and human catechol-*O*-methyltransferase (COMT) was used to first produce vanillic acid from glucose. This was followed by further reduction of vanillic acid to vanillin by whole-cell catalysis of *Neurospora crassa* having the required activity of aromatic carboxylic acid reductase (ACAR) [59]. Thereafter, the whole synthetic vanillin pathway was successfully reconstructed in both *Schizosaccharomyces pombe* and *S. cerevisiae* strains using the *Nocardia iowensis* ACAR, which eliminated the need for a separate whole-cell catalysis step. Moreover, the *C. glutamicum* phosphopantetheinyl transferase was co-expressed in the engineered *S. cerevisiae* strain for activation of ACAR, and endogenous alcohol dehydrogenases in the *S. cerevisiae* host were deleted to prevent reduction of vanillin to vanilly alcohol. In the engineered *S. pombe*, vanillin production was enhanced by introducing the *Arabidopsis thaliana* UDP glycosyltransferase that converts vanillin to *β*-d-glucoside. The use of *β*-d-glucoside could reduce the toxicity caused by vanillin at high concentrations, and the microbially produced *β*-d-glucoside can be subsequently converted back to vanillin through enzymatic conversion [60]. Through deletion of the pyruvate decarboxylase-encoding gene that was suggested by in silico genome-scale metabolic simulation, the vanillin *β*-d-glucoside production could be improved to 500 mg/L in engineered *S. cerevisiae*, which was fivefold higher than that obtained with engineered *S. pombe* [61].

Salicylic acid (2-hydroxybenzoic acid) is widely used in the pharmaceutical industry owing to its analgesic, anti-inflammatory and antipyretic activities [62]. In nature, it is synthesized in plants and bacteria to serve as a signal molecule and a precursor in the production of iron-chelating siderophore, respectively [63]. Even though the salicylic acid biosynthetic pathway has been well studied previously, only recently the highest salicylic acid titer of 11.5 g/L was achieved via CHA in engineered *E. coli*, in which the native PTS was replaced by GalP/Glk system and the downstream glycolytic pathway was blocked to enhance the precursors towards SHK pathway [64]. Apart from *E. coli*, *C. glutamicum* was also engineered to produce salicylic acid to a concentration of 100 mg/L in shake-flask experiments by introducing the *irp9*-encoded bifunctional isochorismate synthase/isochorismate pyruvate lyase from *Yersinia enterocolitica* (Table 1) [65]. The glycosylated form of salicylic acid, salicylate 2-*O*-β-d-glucoside (SAG), was also successfully produced in engineered *E. coli* by the addition of the *A. thaliana* glucosyltransferase to the existing salicylic acid biosynthetic pathway [66]. The SAG titer of 2.5 g/L was achieved by using a co-culture system. In this system, one recombinant *E. coli* strain harboring the upstream salicylic acid pathway was used to produce salicylic acid, which was converted to SAG by the second *E. coli* strain expressing a codon-optimized *A. thaliana* glucosyltransferase gene [67].

*p*-Aminobenzoic acid (PABA) is a widely used compound in pharmaceutical, resin and dye industries, and is currently produced from petroleum-derived toluene. PABA is also a natural metabolite in the folate biosynthesis in plants and bacteria synthesized through a two-step conversion of CHA catalyzed by aminodeoxychorismate (ADC) synthase and 4-amino-4-deoxychorismate (4ADC) lyase [68]. The first microbial production of PABA was achieved in *S. cerevisiae* by overexpressing *ABZ1*-encoded PABA synthase from another *S. cerevisiae* species and also by deleting *ARO7* (encoding CHA mutase) and *TRP3* (encoding indole-3-glycerolphosphate synthase/anthranilate synthase complex) in the aromatic amino acid pathway [28]. Using this engineered *S. cerevisiae* strain, the effects of different carbon sources (glucose, glycerol and glycerol/ethanol mixture) on PABA production were also investigated based on the results obtained from in silico analysis. Among the carbon sources examined, the highest PABA titer and yield reached 215 mg/L and 2.64% mol/mol carbon, respectively, using glycerol/ethanol mixture [69]. Compared to the engineered yeast, engineered *E. coli* harboring *pabA*- and *pabB* (encoding heterodimer complex ADC synthase), *pabC* (encoding 4ADC lyase), and *E. coli aroF*^*fbr*^ (encoding feedback resistant AroF) produced 4.8 g/L of PABA by fed-batch fermentation [70]. The highest PABA titer of 43 g/L was achieved by an engineered *C. glutamicum* harboring the best combination of *Corynebacterium callunae pabAB* and *Xenorhabdus bovienii pabC* in fed-batch culture [71].

Pyrogallol, also known as 1,2,3-trihydroxybenzene, is an important platform chemical used in food, pharmaceutical and polymer industries. Currently it is chemically synthesized from gallic acid in the presence of HCl, where gallic acid is obtained by enzymatic conversion of gallotannin (tannic acid) using tannese [72]. However, this process for commercial production of pyrogallol is limited due to the low yield of gallotannin obtained by plant extraction. The characterization of genes that encode tannese and gallic acid decarboxylase in a number of microorganisms, such as *Streptococcus gallolyticus*, *Lactobacillus plantarum* and *Pantoea agglomerans*, pioneered the research on microbial production of pyrogallol. Starting with DHS in SHK pathway, PCA and gallic acid were sequentially synthesized in engineered *E. coli* by *aroZ*-encoded DHS dehydratase from *K. pneumoniae* and mutated *pobA*-encoded PHBA hydroxylase from *Pseudomonas aeruginosa*, respectively. Using this engineered *E. coli*, the highest reported gallic acid titer of 20 g/L was achieved from glucose in fed-batch fermentation. To produce pyrogallol from gallic acid, this *E. coli* strain was further engineered by introducing *aroY* (encoding PCA decarboxylase) from *K. pneumonia* since the desired enzymatic activity for this conversion was shown by non-oxidative decarboxylase isolated from *Pantoea agglomerans*. However, the pyrogallol production was not detected [73]. In recent study, pyrogallol has been synthesized from CHA via 2,3-DHBA catalyzed by 2,3-DHBA synthase and 2,3-DHBA 1-monoxygenase. *E. coli* overexpressing the native 2,3-DHBA synthase and an efficient 2,3-DHBA 1-monoxygenase (encoded by *nahG*) from *P. putida* produced 201.5 mg/L of pyrogallol from glucose. Pyrogallol production by this recombinant strain was further improved to 1035.8 mg/L by increase of flux to SHK pathway, modular pathway optimization and reduction in pyrogallol autoxidation [75]. Moreover, 1266.4 mg/L of gallic acid was also produced via CHA by engineered *E. coli* harboring a highly efficient mutated PobA from *Pseudomonas aeruginosa* in shake flasks [76].

Quinic acid (QA), commonly used as synthons in pharmaceutical industry, is currently produced from phenol or benzene by petrochemical processes. QA is among the first produced cyclohexane carboxylic acids by engineered microorganisms with the discovery of its catabolism in *K. pneumoniae*. QA production from glucose was first achieved in an engineered *E. coli* expressing the *K. pneumoniae qad* gene encoding QA dehydrogenase [76]. In another study, overexpression of the *E. coli aroE* encoding the native SHK dehydrogenase and knocking out *aroD* encoding DHQ dehydratase resulted in production of 49 g/L QA from glucose by fed-batch culture [77]. Based on the biosynthesized QA in these studies, downstream chemical processes were also developed to produce other important industrial quinone derivatives such as benzoquinone and hydroquinone [77, 78].

Arbutin, a glycosylated hydroquinone in plant metabolism, finds applications in cosmetic industry due to its whitening activity that inhibits tyrosinase-mediated melanogenesis. It also serves as an anti-microbial, anti-inflammatory and anti-oxidant agent. Compared to chemical processes, enzymatic methods for arbutin production is more favorable due to the regioselectivity, mild operation conditions and use of fewer toxic chemicals. In this regard, various plant- and microbial-based approaches have been investigated for enzymatic production of arbutin from hydroquinone using a series of glycosylation enzymes originated from different organisms [78]. De novo biosynthesis of arbutin from glucose was achieved for the first time in an engineered *E. coli* strain expressing the genes encoding the flavin adenine dinucleotide-dependent 4-hydroxybenzoate 1-hydroxylase (MNX1) from *Candida parapsilosis* and uridine phosphate glucose (UPDG)-based hydroquinone glucosyl transferase (AS) from *Rauvolfia serpentine*. Further enhancement of the SHK pathway flux and optimization of initial glucose concentrations improved arbutin production up to 4.2 g/L in shake flasks [79]. In a recent study, the highest reported arbutin titer of 6.79 g/L was achieved by engineered *Pseudomonas chlororaphis* expressing the aforementioned MNX1 and AS genes from the genome [80].

Gastrodin, a phenolic glycoside found in plants, serves as a drug substance based on its sedative, hypnotic, anticonvulsive and neuroprotective activities. It is currently produced by either chemical synthesis or extraction from plants. In nature, some organisms (e.g., *Rhizopus chinensis*) have the ability to transform *p*-hydroxybenzaldehyde to gastrodin [81]. Although the natural gastrodin biosynthesis pathway in plants has not been fully identified, de novo production of gastrodin from glucose has been reported in engineered *E. coli* harboring an artificial metabolic pathway. This synthetic pathway was derived from PHBA, which was further converted by the *Nocardia* carboxylic acid reductase, endogenous alcohol dehydrogenases and an engineered *Rhodiola* glycosyltransferase. The engineered *E. coli* overexpressing the pathway genes produced 545 mg/L of gastrodin [82].

**Fig. 2:**
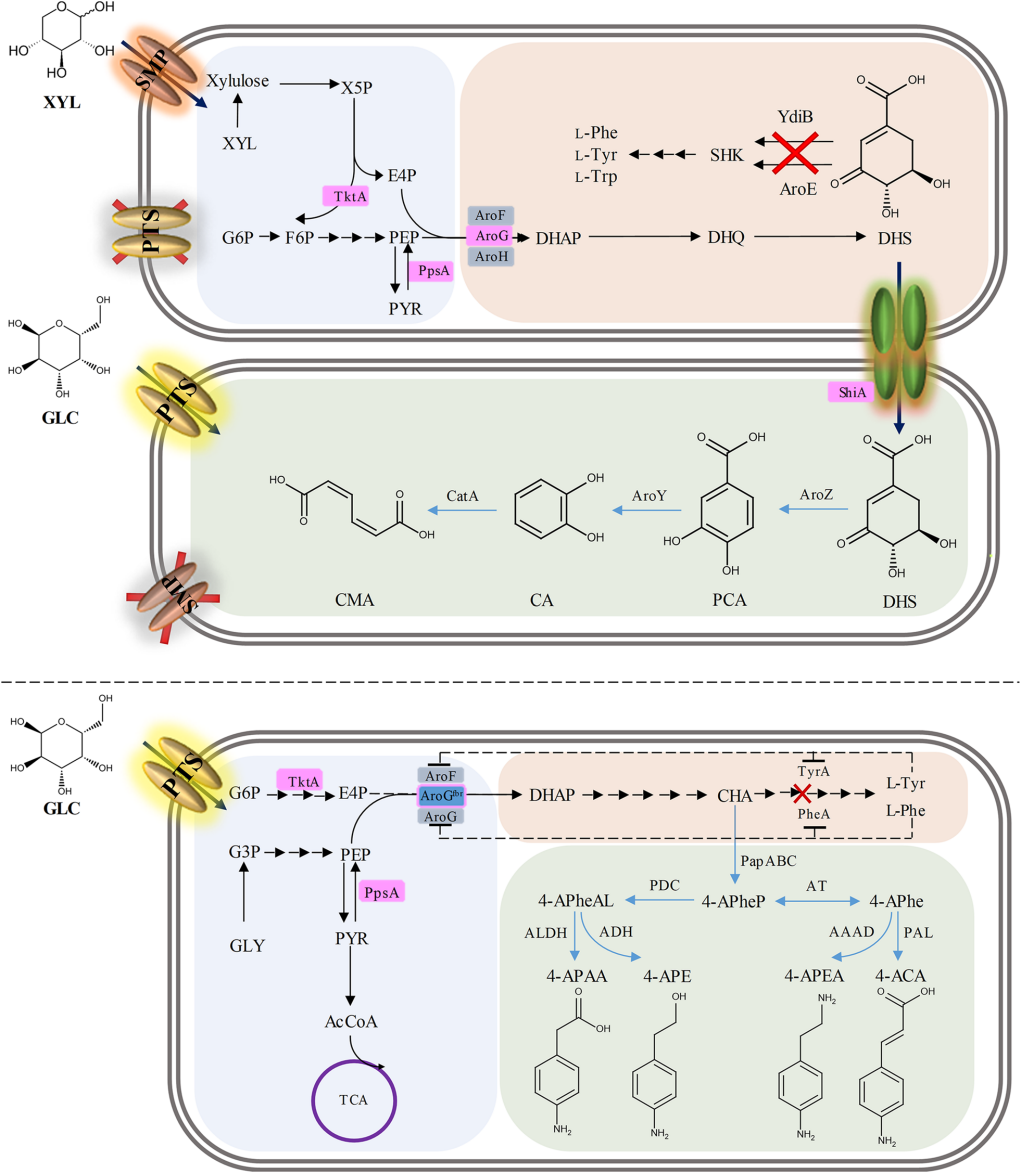
The metabolic engineering approaches to microbial production of SHK pathway derivatives. A microbial co-culture system developed for the biosynthesis of *cis*,*cis*-muconic acid from a mixture of glucose and xylose (shown in the upper panel). Metabolic design for the production of a series of chorismate-derived aromatic amines (shown in the bottom panel). Abbreviations for metabolites: 4-ACA: 4-aminocinnamic acid; 4-APAA: 4-aminophenyl acetic acid; 4-APE: 4-aminophenylethanol; 4-APEA: 4-aminophenylamine; 4-APhe: 4-aminophenylalanine; 4-APheAL: 4-aminophenyl acetaldehyde; 4-APheP: 4-aminophenyl pyruvate; AcCoA: acetyl-CoA; CA: catechol; CHA: chorismate; CMA: *cis*,*cis*-muconic acid; DAHP: 3-deoxy-d-arabino-heptulosonate 7-phosphate; DHQ: 3-dehydroquinate; DHS: 3-dehydroshikimate; E4P: erythrose 4-phosphate; F6P: fructose 6-phosphate; G6P: glucose 6-phosphate; GLC: glucose; GLY: glycerol; PCA: protocatechuic acid; PEP: phosphoenolpyruvate; l-PHE: l-phenylalanine; PYR: pyruvate; SHK: shikimate; TCA: tricarboxylic acid; l-TRP: l-tryptophan: l-TYR: l-tyrosine; X5P: xylose 5-phosphate; XYL: xylose. Abbreviations for enzymes: AAAD: aromatic amino acid decarboxylase; ADH: aldehyde dehydrogenase; ALDH: alcohol dehydrogenase; AroE: shikimate dehydrogenase; AroF: feedback sensitive DHAP synthase; AroG^fbr^: feedback resistance isozyme of DHAP synthase; AroH: chorismate mutase; AT: aminotransferase; AroY: protocatechuate decarboxylase; AroZ: 3-dehydroshikimate dehydratase; CatA: catechol 1,2-dioxygenase; PapA: 4-amino-4-deoxychorismate synthase; PapB: 4-amino-4-deoxychorismate mutase; PapC: 4-amino-4-deoxyprephenate dehydrogenase; PDC: phenylpyruvate decarboxylase; PpsA: phosphoenolpyruvate synthase; ShiA: shikimate transporter, PAL: phenylalanine ammonia lyase; TktA: transketolase; YdiB: Quinate/shikimate dehydrogenase. The inactivated metabolic pathways are indicated by “X”. Dotted lines indicate feedback inhibition. Native metabolic pathways are indicated by black arrows, and non-native pathways are indicated by blue arrows. Multiple metabolic reactions are indicated by sequential arrows. Blue boxes represent feedback inhibition resistant mutants of endogenous enzymes. Pink line or pink boxes represent overexpressed enzymes

A number of aromatic amines have also been produced from glucose by expanding the SHK pathway via CHA in *E. coli* host (Fig. 2). The key step of the artificial biosynthesis of these aromatic amines was the formation of 4-aminophenylpyruvate from CHA by the sequential activities of 4-amino-4-deoxychorismate synthase (encoded by *papA*), 4-amino-4-deoxychorismate mutase (encoded by *papB*) and 4-amino-4-deoxyprephenate dehydrogenase (encoded by *papC*) from *Streptomyces venezuelae* and *Streptomyces pristinaespiralis*. Subsequently, this biosynthetic pathway was branched into 4-aminophenylacetaldehyde and 4-aminophenylalanine by the reactions catalyzed by phenylpyruvate decarboxylase and aminotransferase, respectively. In the last step, 4-aminophenylacetic acid and 4-aminophenylethanol were produced from 4-aminophenylacetaldehyde, while 4-aminocinnamic acid and 4-aminophenylethylamine were produced from 4-aminophenylalanine [83].

## Engineering the biosynthesis pathway of aromatic amino acids and their derivatives

Three aromatic amino acids, l-PHE, l-TYR and l-TRP, are derivatives of CHA and are synthesized through the formation of specific intermediates such as phenylpyruvate, 4-hydroxyphenylpyruvate and anthranilic acid, respectively. Hence, not only aromatic amino acids but also these intermediates play important roles as useful precursors for the production of diverse aromatic acids, alcohols, hydrocarbons, ketones, and aromatic natural compounds (mostly involved in phenylpropanoid pathway). Metabolic engineering for the production of these derivative aromatic products will be described separately according to each of the three aromatic amino acid pathways they are derived from.

### l-PHE pathway derivatives

From l-PHE biosynthetic pathway, the majority of the microbially produced aromatic compounds are derived from two major precursors, phenylpyruvate and l-PHE, which are summarized in Table 2.

**Table 2:** The systems metabolic engineering strategies on phenylalanine pathway for the microbial production of aromatic compounds

Product	Precursor	Carbon source	Host	Titer	Time (h)	Bioprocess strategy	Systems metabolic engineering strategies	References
Phenylpyruvate derivatives								
d-PHG	S-MA	Glucose	*E. coli*	0.036 g/L	24	Batch (shake-flask)	Integrating genes encoding feedback resistant DAHP and bifunctional chorismate mutase/prephenete dehydratase; Deleting three genes in competing pathway using PPA and CHA; Construction 3-step artificial pathway from CHA to d-PHG	[84]
l-PHG	S-MA	Glucose	*E. coli*	–	48	Batch (shake-flask)	Screening better HpgT activity from different sources; Deleting genes in competing pathway using PPA; Construction 3-step artificial pathway from CHA to l-PHG	[86]
S-MA	PPA	Glucose	*E. coli*	0.74 g/L	24	Batch (shake-flask)	Integrating genes encoding feedback resistant DAHP and bifunctional chorismate mutase/prephenete dehydratase; Their overexpression with S-MA; Deleting genes responsible for synthesis of l-TRP, l-TYR and l-PHE in competing pathways using CHA; Construction 3-step artificial pathway from CHA to R-MA	[87]
R-MA	S-MA			1.02 g/L	84			
S-MA	PPA	Glucose	*S. cerevisiae*	0.236 g/L	120	Batch (shake-flask)	Screening better HmaS activity from different microorganisms; Overexpressing genes (encoding pentafunctional protein and feedback two isozyme of resistant DAHP synthase); Deleting genes phenylpyruvate decarboxylase to reduce flux towards Ehrlich pathway	[88]
d-PHE	PPA	Glucose	*E. coli*	1.72 g/L	60	Fed-batch (fermenter, 15 L)	Screening and homology based modeling of d-aminoacid transferase (Dat) from three *Bacillus* species; Constructing artificial d-PHE synthetic operon including each *Dat* gene	[89]
l-PhLA	PPA	Glucose	*E. coli*	1.9 g/L	24	Batch (shake-flask)	Integration feedback-resistant allel of gene involved in SHK and l-PHE amino acid pathway; Deleting genes in competing pathway using CHA. Introducing lactate dehydrogenase with heterologous phenylpyruvate reductase	[91]
Poly-(3HB-co-d-PhLA)	PPA	Glucose	*E. coli*	13.9 g/L	96	Fed-batch (fermenter, 6.6 L)	Identifying and introducing the gene encoding CoA transferase; integrating gene of enzyme responsible conversion from glucose to d-PhLA. Modulating flux under different promoters	[92]
Cinnamic acid	d-PhLA	Glucose	*E. coli*	1.7 g/L	30 (aerobic phase)	Batch coculture (shake-flask)	Integrating phenylpyruvate reductase-coding *pprA from Wickerhamia fluorescens in* d-PhLA producer strain; Integrating phenyllactate dehydratase-coding *fldABCI* from *Clostridium sporogens* in CA producer strain; Performing stepwise process; Optimizing cultivation time of aerobic phase followed by anaerobic one	[105]
Phenylalanine derivatives								
Pinosylvin	Cinnamoyl-CoA	Glucose + CA	*C. glutamicum*	0.121 g/L	72	Batch (shake-flask)	Deleting three gene cluster; Integrating 4CL and STS-coding genes from plant sources; Supplying cerulenin to inhibit activity of enzymes in fatty acid synthesis using malonyl-CoA	[116]
	Cinnamoyl-CoA	Glucose	*E. coli*	0.281 g/L	48	Batch (shake-flask)	Constructing two expression modules; Optimizing expression levels by combining promoter type and copy number in these expression modules; Designing upstream pathway module to direct carbon flux towards precursor; Designing downstream pathway module to produce the molecule of interest	[117]
Chrysin	Pinocembrin	Galactose + acetate	*S. cerevisiae*	0.001 g/L	92	Batch (shake-flask)	Introducing and overexpressing parsley *FSI* gene into biosynthetic pinocembrin pathway	[162]
		Galactose + raffinose		0.002 g/L	92	Batch (shake-flask)	Introducing and overexpressing snapdragon gene (encoding flavone synthase II) and yeast gene (encoding P450 reductase) into biosynthetic pinocembrin pathway	
	Pinocembrin	Glucose + l-PHE	*E. coli*	0.0094 g/L	36	Batch (shake-flask)	Introducing *FNS1* gene from *Petroselinum crispum* into biosynthetic pinocembrin metabolic pathway comprising PAL/TAL, 4CL, CHS, CHI and ACC (encoded by *dtsR1* and *accBC*)	[164]
Cinnamic acid	l-PHE	Glucose + casaminoacids	*E. coli*	6.9 g/L	86	Fed-batch (fermenter, 2 L)	Screening appropriate promoter to express *Streptomyces maritimus PAL* gene; Investigating the effect of different supplements in feeding solution on pH-stat fed-batch fermentation	[106]
Styrene	CA	Glucose	*E. coli*	0.26 g/L	29	Batch (shake-flask)	Introducing genes encoding plant phenylalanine ammonia lyase and yeast trans-cinnamate decarboxylase into l-PHE overproducer strain lacking multiple genes and expressing genes encoding feedback resistant enzymes in SHK pathway	[107]
Pinocembrin	Cinnamyl-CoA	Glucose	*E. coli*	0.710 g/L	36	Batch (shake-flask)	Overexpressing malonate carrier- and malonate synthase-coding genes involved in the malonate assimilation pathway; Repressing activity of two genes in fatty acid pathway by the addition of cerulenin	[159]
Galangin	Pinocembrin	Glucose + l-PHE	*E. coli*	0.001 g/L	36	Batch (shake-flask)	Introducing *F3H* and *FLS* gene from *Citrus* species into biosynthetic pinocembrin metabolic pathway comprising PAL/TAL, 4CL, CHS, CHI and ACC (encoded by *dtsR1* and *accBC*)	[164]

d-Phenylglycine (d-PHG) serves as a precursor in the production of semi-synthetic antibiotics including cephalosporin and penicillin [84], and it is either chemically produced from phenol or enzymatically synthesized by resolution of racemic mixtures originating from fine chemicals [85]. Microbial biosynthesis of d-PHG from glucose has been achieved through a three-step artificial pathway after phenylpyruvate, which consisted of the successive formation of *S*-mandelate, phenylglyoxylate and d-PHG catalyzed by *hmaS*-encoded 4-hydroxymandelate synthase of *Amycolatopsis orientalis*, *hmo*-encoded 4-hydroxymandelate oxidase of *Streptomyces coelicolor* and *hpgAT*-encoded stereoinverting hydroxyphenylglycine aminotransferase of *P. putida*, respectively. The engineered *E. coli* expressing these enzymes and with deletions of the aspartate aminotransferase-encoding *aspC* and aromatic amino acid aminotransferase-encoding *tyrB* genes produced 102 mg d-PHG per gram dry cell weight (DCW) from glucose [84]. Moreover, l-phenylglycine (l-PHG), a precursor in the production of several antibiotics and taxol, was also produced by replacing the HpgAT in the d-PHG pathway with l-4-hdroxyphenylglycine aminotransferase (encoded by *hpgT*) from *A. orientalis* and *S. coelicolor*. The engineered *E. coli* harboring these heterologous enzymes produced 51.6 mg/g DCW of l-PHG from glucose [86].

Mandelic acid is one of the aromatic fine chemicals primarily used in pharmaceutical industry and for resolution of racemic alcohols and resins. Compared to chemical methods, enzymatic synthesis of mandelic acid is more attractive as it can afford stereoisomers of mandelic acid. An engineered *E. coli* strain expressing the *A. orientalis* HmaS and with deleted competing pathways produced 0.74 g/L of *S*-mandelate from glucose. By further expressing the *S. coelicolor* Hmo and d-mandelate dehydrogenase (encoded by *dmd*) from *Rhodotorula graminis* in the *S*-mandelate-producing *E. coli*, 0.68 g/L of *R*-mandelate was produced from glucose [87]. By replacing the *A. orientalis* HmaS with a more efficient isozyme from *Nocardia uniformis* in an engineered *S. cerevisiae*, 236 mg/L of *S*-mandelate was produced from glucose [88].

Similar to d-PHG, d-phenylalanine (d-PHE) is also a building block in the production of semi-synthetic antibiotics such as penicillin and cephalosporin. As a l-stereoisomer, l-PHE is synthesized from phenylpyruvate by l-PHE aminotransferase, however, such an aminotransferase with d-stereoisomer activity (i.e., d-PHE aminotransferase) for producing d-PHE is still lacking. Therefore, microbial biosynthesis of d-PHE has been achieved by using the d-amino acid aminotransferase (Dat) in *Bacillus* species instead. Three Dats from *Bacillus subtilis*, *Bacillus licheniformis* and *Bacillus amyloliquefaciens* were compared through 3D structural modeling and experimental screening. When the best-performing Dat from *B. subtilis* was expressed in an l-PHE-producing *E. coli*, 1.72 g/L of d-PHE was produced from glucose in bioreactor fermentation [89].

**Fig. 3:**
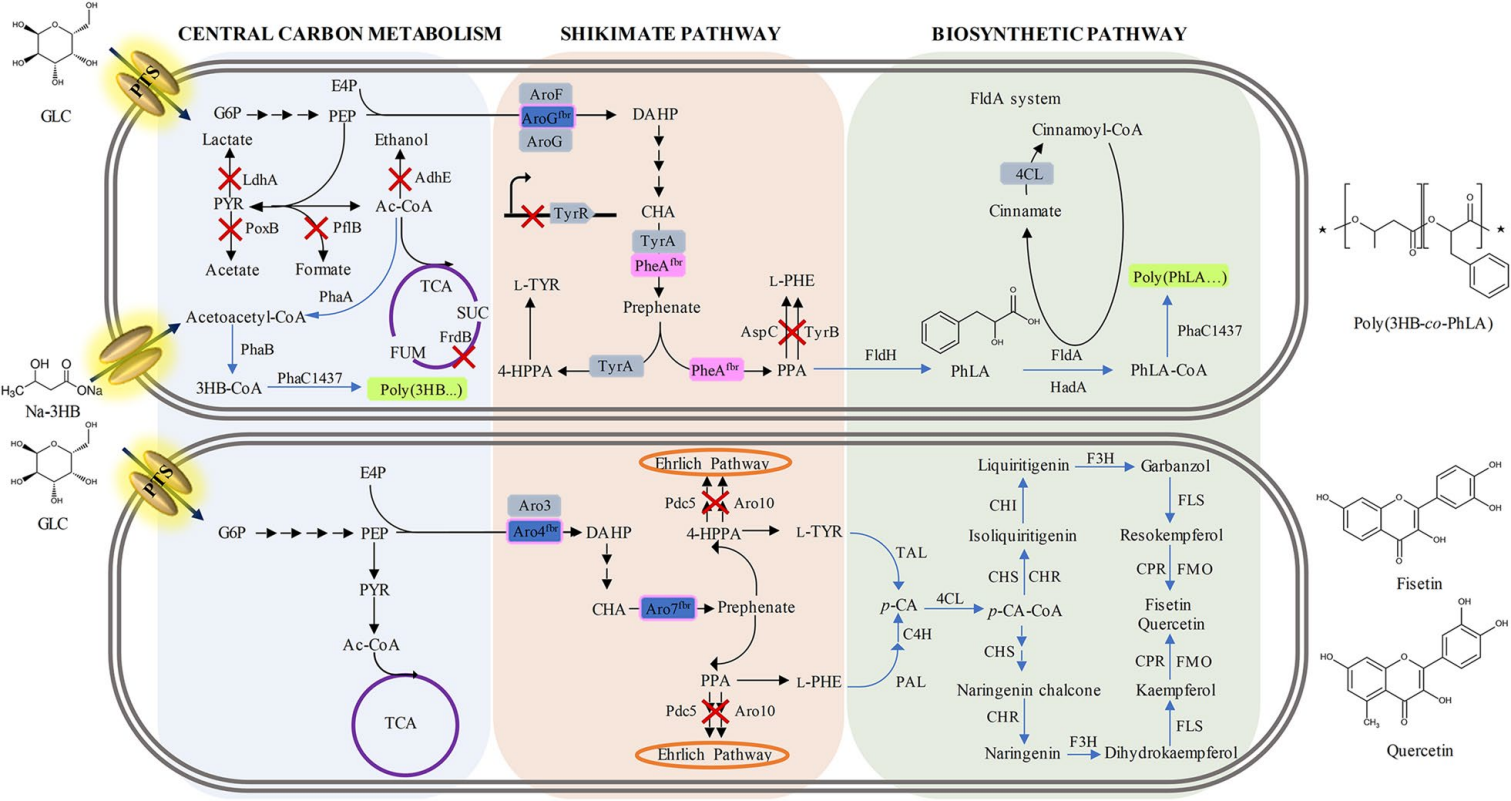
The metabolic engineering approaches to microbial production of aromatic amino acid derivatives. A systematic study for the de novo microbial production of aromatic-containing polyester derived from phenylalanine pathway (shown in the upper panel). De novo biosynthesis of the aromatic plant natural products, fisetin and quercetin, derived from phenylalanine and tyrosine pathways (shown in the bottom panel). Abbreviations for metabolites: 3HB-CoA: 3-hydroxybutyryl-CoA; 4-HPPA: 4-hydroxyphenylpyruvic acid; Ac-CoA: acetyl-CoA; CHA: chorismate; DAHP: 3-deoxy-d-arabino-heptulosonate 7-phosphate; E4P: erythrose 4-phosphate; FUM: fumarate; G6P: glucose 6-phosphate; Na-3HB: sodium 3-hydroxybutyrate; *p*-CA: *p*-coumaric acid; *p*-CA-CoA: *p*-coumaroyl-CoA; PEP: phosphoenolpyruvate; l-PHE: l-phenylalanine; PhLA: d-phenyllactate; PhLA-CoA: d-phenyllactatyl-CoA; PPA: phenylpyruvate; PYR: pyruvate; SUC: succinate; l-TYR: l-tyrosine. Abbreviations for enzymes: 4CL: 4-coumaric acid CoA ligase; AdhE: aldehyde-alcohol dehydrogenase; Aro3: 3-deoxy-7-phosphoheptulonate synthase; Aro4: feedback resistance isozyme of phosphote-3-dehydro-3-deoxyheptonate aldolase; Aro7: feedback resistance isozyme of chorismate mutase; Aro10: transaminated amino acid decarboxylase; AroF: feedback sensitive DHAP synthase; AroG^fbr^: feedback resistance isozyme of DHAP synthase; AspC: aspartate aminotransferase; C4H: cinnamic acid decarboxylase; CHI: chalcone isomerase; CHR: chalcone reductase; CHS: chalcone synthase; CPR: cytochrome P450 reductase; F3H: flavanone 3-hydroxylase; FldA: cinnamoyl-CoA:phenyllactate CoA-transferase; FldH: d-phenyllactate dehydrogenase; FLS: flavanol synthase; FMO: flavonoid 3′-monooxygenase; FrdB: fumarate reductase iron–sulfur subunit; HadA: isocaprenoyl-CoA:2-hydroxyisocaproate CoA-transferase; LdhA: d-lactate dehydrogenase; PAL: phenylalanine ammonia lyase; PflB: formate acetyltransferase 1; Pdc5: pyruvate decarboxylase isozyme; PhaA: acetyl-CoA acetyltransferase; PhaB: acetoacetyl-CoA reductase; PhaC: poly(3-hydroxyalkanoate) polymerase subunit; PhaC1437: variant of PhaC; PheA^fbr^: feedback resistance isozyme of bifunctional chorismate mutase/prephenete dehydratase; PoxB: pyruvate dehydrogenase; TAL: tyrosine ammonia lyase; TyrA: prephenate dehydrogenase; TyrB: tyrosine aminotransferase; TyrR: transcriptional regulatory protein 2. The inactivated metabolic pathways are indicated by “X”. Native metabolic pathways are indicated by black arrows, and non-native pathways are indicated by blue arrows. Sequential arrows indicate multiple metabolic reactions. Orange eclipse displays other native metabolism. Blue boxes represent feedback inhibition resistant mutants of endogenous enzymes. Pink line or pink boxes represent overexpressed enzymes. Green boxes represent aromatic polymers

Phenyllactic acid (PhLA) is a potential monomer that can be used in the production of sustainable polyesters by chemical polymerization with other bio-based monomers [90]. Both d-PhLA and l-PhLA have been biosynthesized through one-step reduction of phenylpyruvate. An l-PHE-overproducing *E. coli* expressing the *pprA*-encoded phenylpyruvate reductase from *Wickerhamia fluorescens* produced 29 g/L of d-PhLA from glucose in bioreactor fermentation with optimized conditions [91]. In the same *E. coli* strain, l-PhLA was also produced from glucose by replacing PprA with the *ldhA*-encoded lactate dehydrogenase from *Pediococcus acidilactici* [91]. For advanced applications, d-PhLA was co-polymerized with 3-hydroxybutyrate to make novel aromatic polyesters by engineered *E. coli* through one-step fermentation. In achieving this, the pivotal steps were the choices of a suitable CoA-transferase and an engineered polyhydroxyalkonate synthase (Fig. 3) [92].

Phenylpropanoids constitute a response system in plant metabolism to protect plants against various changes in their environment. Also, they are important precursors in the production of various chemicals used in food, pharmaceutical and cosmetic industries. Biosynthesis of phenylpropanoids starts with the formation of cinnamic acid, which is a key branch point leading to the biosynthesis of various other phenylpropanoids [93]. Metabolic engineering of phenylpropanoids has been focused on cinnamic acid and its derivatives including several secondary metabolites such as flavonoids, stilbenoids, coumarins, among other phenylpropanoids [7].

Cinnamic acid (CA) is chemically synthesized either through the Perkin reaction by heating benzaldehyde and acetic anhydride with sodium acetate [94] or through the Knoevenagel condensation of malonic acid and aromatic aldehydes [95]. In plants, l-PHE is catalyzed by phenylalanine ammonia-lyase (PAL) to yield *trans*-CA [96]. While some PALs such as the yeast *Rhodosporidium toruloides* PAL possess the alternative capability to convert l-TYR to *p*-hydroxycinnamic acid in addition to converting l-PHE to CA [97], the *Streptomyces maritimus* PAL shows a high substrate selectivity towards l-PHE, and it has been used for the biosynthesis of CA in engineered *E. coli* [98]. Expression of the *R. toruloides* PAL in a solvent-tolerant and mutated *P. putida* strain with enhanced supply of the precursor l-PHE led to production of 0.741 g/L CA [99]. Moreover, *Streptomyces lividans*, well-known for its superior biomass-assimilating ability, was also engineered to produce CA by expressing the *S. maritimus* PAL, which resulted in 210, 450, 460, 300 and 130 mg/L of CA in shake-flask cultures from glucose, glycerol, raw starch, xylose and xylan, respectively [100]. By using 30 g/L of cello- and xylo-oligosaccharide as carbon sources, CA production levels by engineered *S. lividans* reached 490 and 450 mg/L, respectively [101]. The engineered *E. coli* strain expressing the PAL/TAL from *Rhodotorula glutinis* or *A. thaliana* was evaluated for CA production in different carbon sources (i.e., glucose, xylose or arabinose, as well as a mixture of these three sugars mimicking the lignocellulosic hydrolysate). As a result, *E. coli* carrying the *A. thaliana* TAL/PAL using arabinose produced the highest CA titer [102]. In addition to the conventional approach using TAL/PAL, microbial CA production has also been achieved by the *fldABCI*-encoded phenyllactate dehydratase from *Clostridium sporogenes* [103–105]. Under optimized oxygen conditions, two-step bioprocess approach enabled 1.7 g/L of CA through stepwise production of d-PhLA and CA in *E. coli* strain expressing W. fluorescens *pprA* and *C. sporogenes fldABCI*, respectively [105]. In a most recent study, the highest reported CA titer of 6.9 g/L was achieved by engineered *E. coli* harboring a PAL in a fed-batch culture [106].

Styrene is a petroleum-derived chemical produced from dehydrogenation of ethylbenzene, and it is also formed by non-oxidative decarboxylation of CA via microbial biosynthesis [107]. Although the bioconversion of CA to styrene was shown almost a century ago [108], the first artificial biosynthetic pathway for styrene production from glucose was only established recently, through deamination of l-PHE by the *A. thaliana PAL2*-encoded lyase followed by decarboxylation of CA to styrene by the *S. cerevisiae FDC1*-encoded ferulate decarboxylase. While phenylacrylate decarboxylase from *S. cerevisiae* was also identified to be able to decarboxylate CA, the higher styrene titer of 260 mg/L was achieved through the use of PAL2 and FDC1 [107]. A co-culture system of *S. lividans* strains expressing the *S. cerevisiae* FDC1 and *S. maritimus* PAL was developed to produce styrene from different carbon sources such as glucose, cellobiose or xylo-oligosaccharide, which however did not lead to an increase in styrene production [109].

Hydroxycinnamic acids are a group of CA derivatives, which mainly consist of *p*-hydroxycinnamic acid (*p*-coumaric acid), 3,4-dihydroxycinnamic acid (caffeic acid) and 4-hydroxy-3-methoxycinnamic acid (ferulic acid) [110]. There is a sequential bioconversion from *p*-coumaric acid to ferulic acid through caffeic acid. These hydroxycinnamic acids will be described in the following section as they are derived from the l-TYR biosynthesis pathway.

Among various flavonoids, pinocembrin as a plant secondary metabolite is an important substance used in food and pharmaceutical industries thanks to its anti-inflammatory, anti-microbial, anti-cancer and neuroprotective activities [111]. In plant metabolism, pinocembrin is a branch point for the biosynthesis of several other flavonoids including galangin, dihydroflavonol and chrysin, through enzymatic modifications in its core structure. Microbial production of pinocembrin and related flavonoids will be discussed later as both l-PHE and l-TYR biosynthesis pathways are involved in producing these molecules.

Among non-flavonoid phytochemicals in plants, stilbenes (stilbenoids) are polyphenolic secondary metabolites, which come to the fore with the 1,2-diphenylethylene nucleus. Similar to flavonoids, stilbenes are synthesized to defense plants against various environmental stresses such as wounding, fungal infection, UV radiation, etc. They are also important drug substances because of their anti-cancer, anti-inflammatory and anti-obesity activities [112, 113]. Well-known stilbenes produced by microbial systems are resveratrol, pterostilbene, piceatannol, pinosylvin, etc. The natural biosynthesis of stilbenes follows a similar pathway to those of flavonoids and phenylpropanoids through the intermediary formation of CoA esters, i.e., cinnamoyl-CoA and *p*-coumaroyl-CoA. These aromatic CoA esters, cinnamoyl-CoA and *p*-coumaroyl-CoA, are condensed with three malonyl-CoA units by stilbene synthase (STS) to produce the stilbene backbone, followed by the formation of pinosylvin and resveratrol, respectively. In a similar fashion, t-piceatannol is also produced using caffeic acid as a starter unit. Additionally, various more complex stilbenes are synthesized through modifications of the stilbene backbone using reactions including glycosylation, methylation, oligomerization and prenylation [114]. Microbial production of pinosylvin from glucose was accomplished in an engineered *E. coli* harboring PAL, 4-coumaric acid CoA-ligase (4CL) and STS. Through the use of an evolved STS with higher catalytic activity as well as the addition of cerulenin for increased malonyl-CoA pool and l-PHE for precursor supply, the pinosylvin titer was increased up to 91 mg/L [115]. Engineered *C. glutamicum* expressing the same set of enzymes responsible for pinosylvin synthesis was also developed, which produced 121 mg/L pinosylvin from CA supplemented [116]. Moreover, the highest reported pinosylvin titer of 281 mg/L was achieved by an engineered *E. coli* harboring the pinosylvin synthetic pathway optimized through a rational modular approach [117].

### l-TYR pathway derivatives

The l-TYR biosynthesis pathway starts with the formation of 4-hydroxyphenylpyruvic acid (4-HPPA) from SHK pathway. Thus, microbial production of 4-HPPA and l-TYR derivatives are dependent on this pathway (Table 3). Despite the biosynthesis of many l-TYR derivatives, only a few 4-HPPA derivatives have been biosynthesized in engineered microbes.

**Table 3:** The system metabolic engineering strategies on tyrosine pathway for the microbial production of aromatic compounds

Product	Precursor	Carbon source	Host	Titer	Time (h)	Bioprocess strategy	Systems metabolic engineering strategies	References
4-HPPA derivatives								
Salvianic acid A	4-HPPA	Glucose	*E. coli*	7.1 g/L	70	Fed-batch (shake-flask)	Introducing heterologous gene for glucose transport; deleting genes responsible for PEP and precursor consumption, and transcriptional repression; Overexpressing genes in PP and SHK pathway; Replacing genes encoding feedback-sensitive prephenate dehydrogenase and DAHP synthase with resistant ones	[118]
		Glucose	*E. coli*	5.6 g/L	60	Fed-batch (Fermentor, 5 L)	λ Red homologous recombination of the modules mentioned in previous study along with similar metabolic engineering strategies	[119]
Rosmarinic acid	4-HPPA	Glucose	*E. coli*	0.13 g/L	30	Batch (shake-flask)	Inserting LDH, HPAH and RAS-coding genes in l-TYR over-producing strain	[123]
L-TYR derivatives								
Tyrosol	4-HPPA	Glucose	*E. coli*	0.573 g/L	48	Batch (shake-flask)	Introducing gene encoding pyruvate decarboxylase (PDC) from *S. cerevisiae*; deleting genes in competing pathways to modulate flux towards 4-HPPA	[125]
	l-TYR	Tyrosine	*E. coli*	1.203 g/L	20	Whole-cell	Introducing aminotransferase gene into biosynthetic tyrosol pathway	
	l-TYR	Glucose	*E. coli*	0.531 g/L	36	–	Introducing plant gene encoding aromatic amino acid synthase that enables one-step conversion of l-TYR to 4-HPAA	[126]
Hydroxytyrosol	l-TYR	Glucose	*E. coli*	0.268 g/L	30	Batch	Introducing genes encoding feedback-resistant DAHP synthase and prephenate dehydrogenase; Deleting genes responsible for transcriptional repression; Overexpressing genes to modulate carbon flux to DAHP	[129]
Salidroside	Tyrosol	Glucose/xylose	*E. coli*–*E. coli*	6.03 g/L	129	Fed-batch co-culture (fermentor, 5 L)	Constructing two strains. Deleting genes responsible for PEP consumption, transcriptional repression, competing pathways, GLC consumption in tyrosol producer upstream strain. Deleting genes responsible for XYL and UDP-GLC consumption, and TYR synthesis in downstream salidroside producer strain. Overexpressing genes in PP pathway.	[127]
*p*-Coumaric acid	l-TYR	Glucose	*S. cerevisiae*	1.93 g/L	72	Fed-batch (well-plate)	Overexpressing genes in SHK pathway a heterologous *TAL* gene; Deleting genes in competing pathways	[135]
Caffeic acid	*p*-coumaric acid	Glucose/glycerol + PHCA	*E. coli*	2.8 g/L	24	Whole-cell biocatalysis	Engineering P450 hydroxylase for desired activity to convert *p*-coumaric acid to caffeic acid	[141]
*p*-HS	*p*-coumaric acid	Glucose	*P. putida*	17.6 g/L	~ 64	Two-phase decanol system	Deleting gene encoding feruloyl-coenzyme A competes for *p*-coumarate intermediates; Introducing two heterologous genes, *pal* and *pdc*	[152]
Naringenin	Coumaroyl-CoA	Glucose	*E. coli*	0.474 g/L	24	Batch (shake-flask)	According to genome scale model, deleting/down-regulating genes in TCA cycle; overexpressing genes in CCM and malonate assimilation pathway	[160]
Kaempferol	Naringenin	Glucose	*S. cerevisiae*	0.026 g/L	72	Fed-batch (well-plate)	Inserting seven heterologous genes originated from different plants	[170]
Apigenin	Naringenin	Glucose + l-TYR	*E. coli*	0.013 g/L	36	Batch (shake-flask)	Introducing *FNS1* gene from *Petroselinum crispum* into biosynthetic naringenin pathway comprising PAL/TAL, 4CL, CHS, CHI and ACC (encoded by *dtsR1* and *accBC*)	[164]
Luteolin	Apigenin	Raffinose + caffeic acid	*S. cerevisiae*	0.002 mg/L	92	Batch (shake-flask)	Expressing five heterologous genes from plants; Investigating efficiency of native and heterologous CPR	[162]
Fisetin	Resokaempferol	Glucose	*S. cerevisiae*	0.002 mg/L	72	Fed-batch (well-plate)	Inserting nine codon optimized genes originated from plants	[170]
Quercetin	Eriodictyol	Glucose	*S. cerevisiae*	0.020 g/L	72	Fed-batch (well-plate)	Inserting eight heterologous genes originated from different plants	[170]
Resveratrol	Coumaroyl-CoA	Glucose	*S. cerevisiae*	0.415 g/L	~ 35	Fed-batch (fermenter, 1 L)	Inserting three heterologous genes originated from plant; Overexpressing genes encoding feedback resistant enzymes in SHK pathway and gene encoding post-translational deregulated acetyl-coA carboxylase; Multiple integration of pathway genes	[174]
		Ethanol		0.531 g/L	~35			
Eriodictyol	Caffeoyl-CoA	Glucose	*E. coli*	0.054 g/L	36	Batch (shake-flask)	Overexpressing malonate carrier- and malonate synthase-coding genes involved in the malonate assimilation pathway; Repressing activity of two enzymes in fatty acid pathway by the addition of cerulenin; Introducing four heterologous genes	[159]

4-Hydroxyphenyllactic acid (4-HPLA) is known for its anti-fungal activity against several species of filamentous fungi, and it is naturally produced by *Bifidobacteria* and *Lactobacilli*. The biosynthesized 4-HPLA via l-TYR in engineered microbes were used as a precursor to further produce other compounds such as aromatic polyesters [92] and salvianic acid A [118].

Salvianic acid A (also known as danshensu and 3-(3′,4′-dihydroxyphenyl)-2-hydroxypropanoic acid) is a natural compound in the plant *Salvia miltiorrhiza*, and it is attractive because of its anti-oxidant and therapeutic activities. Even though the natural biosynthesis pathway of salvianic acid A has not been fully revealed, an artificial biosynthetic pathway for salvianic acid A has been constructed in *E. coli* through a two-step bioconversion from 4-HPPA catalyzed by *Lactobacillus pentosus* d-lactate dehydrogenase (encoded by mutated *d*-*ldh*) and *E. coli hpaBC*-encoded hydroxylase complex [118]. Co-expression of these two enzymes resulted in two different metabolic pathways characterized by particular intermediates 4-HPLA and 3,4-dihydroxyphenylpyruvate, due to the different sequence of the reactions catalyzed (Fig. 1). To enhance flux towards 4-HPPA, a modular approach was applied by constructing three expression plasmids harboring the pathway genes within modules such as *aroG*^fbr^-*tyrA*^fbr^-*aroE*, *ppsA*-*tktA*-*glk* and *hpaBC*-*d*-*ldh*^*Y52A*^, along with deletion of *ptsG*, *pykA*, *pheA* and *tyrR*. As a result, the highest reported salvianic acid A titer of 7.1 g/L was achieved in shake-flask cultures [118]. Furthermore, these gene expression modules were integrated into the *E. coli* chromosome to overcome the issues with plasmid instability and requirement of expensive antibiotics, which however resulted in lower salvianic acid A titer [119].

Rosmarinic acid (RA) is an ester of 3,4-dihydroxyphenyllactic acid and caffeic acid, and is biosynthesized from l-PHE and l-TYR in several plants such as *Rosmarinus officinalis*, *Salvia officinalis* and *Perilla frutescens* [120]. RA and its derivatives such as isorinic acid and lithospermic acid are promising compounds in pharmaceutical and nutraceutical industries owing to their anti-oxidant, anti-bacterial, anti-inflammatory, anti-allergic, neuroprotective and anti-viral activities [121]. According to the elucidated metabolic pathway in *Coleus blumei*, RA is synthesized from two intermediate precursors 4-coumaroyl-CoA and 4-hydroxyphenyllactic acid (4-HPLA) derived from l-PHE and l-TYR, respectively. To obtain 4-coumaroyl-CoA, l-PHE is catalyzed sequentially by PAL, cinnamic acid 4-hydroxylase (C4H) and 4CL, while 4-HPLA is produced from l-TYR by tyrosine aminotransferase and hydroxyphenylpyruvate reductase (HPPR). Thereafter, 4-coumaroyl-4′-hydroxyphenyllactate is yielded from 4-coumaroyl-CoA and 4-HPLA by RA synthase (RAS). In the last step, the above intermediate is hydroxylated by a cytochrome P450 monooxygenase to synthesize RA [122, 123]. Different from this natural RA pathway, an artificial biosynthetic pathway was also constructed in a l-TYR-overproducing *E. coli* by expressing the *A. thaliana* 4CL, *E. coli* 4-hydroxyphenylacetate 3-hydroxylase (encoded by *hpaBC*), *L. pentosus* d-lactate dehydrogenase (encoded by *d*-*ldh*^Y52A^) and *C. blumei* RAS, as well as supplementing caffeic acid, which successfully produced 130 and 252 mg/L of RA and isorinic acid, as well as 55 mg/L of a novel compound caffeoyl-phenyllactate [123].

A group of phenolic compounds called phenylethanoids including tyrosol (*p*-hydroxyphenylethanol), hydroxytyrosol and salidroside are derived from l-TYR pathway. Tyrosol and hydroxytyrosol are naturally present in olive oil and wine, and salidroside is found in the plant *Rhodila rosea*. These phenylethanoids are well-known for their anti-oxidant activities, and they are industrially produced by chemical syntheses. The natural biosynthesis pathway for tyrosol via l-TYR in *S. cerevisiae* has been revealed and named as Ehrlich pathway. More specifically, l-TYR is sequentially converted to 4-HPPA, 4-hydroxyphenylacetaldehyde (4-HPAA) and tyrosol catalyzed by aminotransferase, 4-HPPA decarboxylase and alcohol dehydrogenase (ADH), respectively [124]. This yeast tyrosol pathway was established in an engineered *E. coli* strain with double deletion of *pheA* (encoding prephenate dehydratase) and *feaB* (encoding phenylacetaldehyde dehydrogenase). Also, the *S. cerevisiae ARO10* and *ARO8* genes encoding phenylpyruvate decarboxylase (PDC) and aminotransferase, respectively, were additionally expressed. Under optimized culture condition, the engineered *E. coli* expressing *ARO10* produced 0.537 g/L of tyrosol from glucose. By introducing *ARO8* to this engineered *E. coli*, 1.203 g/L of tyrosol was produced from 1.812 g/L of l-TYR [125]. In a recent study, a recombinant *E. coli* expressing a gene encoding the aromatic acetaldehyde synthase (AAS) that is able to convert l-TYR to 4-HPAA in a single step was constructed. This engineered strain produced 531 mg/L tyrosol from glucose. Two more products could be produced by co-expressing additional genes in the above strain: 208 mg/L of hydroxytyrosol by co-expressing the *hpaBC* gene encoding HpaBC and 288 mg/L of salidroside by co-expressing a gene encoding uridine diphosphate-dependent glycosyltransferase (UGT) UGT85A1 [126]. Furthermore, *E. coli*–*E. coli* co-culture system was developed by separating biosynthetic pathways of tyrosol and salidroside, which led to production of 6.03 g/L of salidroside from a glucose/xylose mixture [127].

An alternative tyrosol pathway was also established by expressing the genes encoding the l-TYR decarboxylase (TYDC) from *Papaver somniferum*, tyramine oxidase from *Micrococcus luteus* and *E. coli* endogenous ADHs in *E. coli* via the formation of tyramine and 4-HPAA. Further deletion of *feaB* involved in the by-product 4-hydroxyphenylacetate formation increased tyrosol production to a titer of 68 mg/L from glucose [128]. Additional co-expression of *E. coli* 4-hydroxyphenylacetate 3-monooxygenase in an engineered *E. coli* expressing this synthetic tyrosol pathway led to production of 268.3 mg/L of hydroxytyrosol from glucose [129]. In addition, hydroxysalidroside was produced from hydroxytyrosol at a conversion yield of approximately 45% by the engineered *E. coli* expressing a selected UGT [129].

Microbial production of hydroxytyrosol was also achieved using an alternative biosynthetic pathway comprising the l-TYR hydroxylase (TH) from mouse, l-DOPA decarboxylase from pig and tyramine oxidase from *M. luteus*. In this strain, l-TYR was converted to hydroxytyrosol via l-3,4-dihydroxyphenylalanine (l-DOPA), dopamine and 3,4-dihydroxyphenylacetaldeyhde. This engineered strain was able to produce 0.08 mM hydroxytyrosol from glucose. It was also reported that *E. coli* endogenous cofactor tetrahydromonapterin (MH4) could serve as an alternative cofactor to the eukaryote-specific cofactor tetrahydrobiophterin (BH4) required by TH [130].

*p*-Hydroxycinnamic acid (also known as *p*-coumaric acid), is synthesized by the *o*-hydroxylation of CA catalyzed by a CO-sensitive cytochrome P450 hydroxylase in plants [131]. Alternatively, *p*-coumaric acid is produced through the direct deamination of l-TYR by TAL in several microorganisms such as *Rhodobacter capsulatus* and *Rhodobacter sphaeroides* [132]. Enzymes responsible for this deamination reaction are classified into (1) TAL that solely converts l-TYR to *p*-coumaric acid and (2) PAL/TAL that catalyzes not only the conversion of l-TYR to *p*-coumaric acid but also conversion of l-PHE to CA [97]. Due to the metabolic burden caused by the P450 hydroxylase, TAL-based approach is more desirable to synthesize *p*-coumaric acid in a microbial host. An engineered *P. putida* strain was constructed to produce *p*-coumaric acid from glucose via l-TYR. This engineered strain was further optimized through expression of a *m*-fluoro-dl-phenylalanine-resistant mutant PAL, deletion of the feruloyl-CoA synthetase gene (*fcs*) involved in *p*-coumaric acid degradation and construction of a l-PHE-auxotrophic mutant to avoid the accumulation of CA. Consequently, 1.7 g/L of *p*-coumaric acid was produced from glucose in fed-batch culture [133]. Apart from pathway engineering, the choice of a TAL enzyme having higher activity and substrate specificity towards l-TYR is a key strategy to solve the bottleneck in the production of *p*-coumaric acid. To this end, TALs or PALs from diverse sources were investigated to identify the most efficient ones for *p*-coumaric acid synthesis in different hosts such as *E. coli*, *Lactoccous lactis* and *S. cerevisiae*. While the PALs from *Brevibacillus laterosporus*, *Dictyostelium discoideum* and *Physcomitrella patens* showed exclusive activity towards l-PHE, the TALs from *Herpetosiphon aurantiacus* and *Flavobacterium johnsoniaeu* exhibited higher reported substrate specificity towards l-TYR [134]. By expressing the *F. johnsoniaeu* TAL gene in an engineered *S. cerevisiae* strain lacking in genes encoding phenylpyruvate decarboxylase and pyruvate decarboxylase involved in competing pathways and overexpressing the genes encoding feedback-resistant DAHP synthase, CHA mutase and *E. coli* SHK kinase II, the highest reported *p*-coumaric acid titer of 1.93 g/L was achieved from glucose in fed-batch culture [135].

3,4-Dihydroxycinnamic acid (caffeic acid) is a common phytochemical synthesized in plants together with ferulic acid. Caffeic acid and its phenethyl ester (CAPE) are used in the pharmaceutical and cosmetic industries due to their anti-oxidant, anti-aging and anti-carcinogenic activities. Despite its high demand on the market, caffeic acid is currently obtained by the extraction from plants [135] and its ester by the subsequent chemical synthesis [136]. In plants, caffeic acid is synthesized through three sequential reactions converting l-PHE to CA, *p*-coumaric acid, and finally to caffeic acid, which are catalyzed by PAL, cytochrome P450-dependent monooxygenase (C4H) and *p*-coumarate 3-hydroxylase (C3H), respectively [137]. An alternative biosynthetic pathway of converting l-TYR to caffeic acid via *p*-coumaric acid has also been identified in the actinomycete *Saccharothrix espanaensis*, which is composed of two reactions catalyzed by TAL (encoded by *sam8*) and C3H (encoded by *sam5*) [138]. The recombinant *E. coli* expressing the *S. espanaensis sam8* and *sam5* genes successfully produced caffeic acid from glucose [139]. This two-step caffeic acid pathway has also been established by using alternative enzymes. The *E. coli* 4-hydroxyphenylacetate 3-hydroxylase encoded by the *hpaBC* operon was shown to have a promiscuous activity of converting *p*-coumaric acid to caffeic acid. Co-expression of this *hpaBC* operon with the *R. capsulatus* TAL gene in a l-TYR-overproducing *E. coli* led to production of 50.2 mg/L of caffeic acid from glucose in shake flasks [140]. In addition, a bacterial P450 hydroxylase CYP199A2 from *Rhodopseudomonas palustris* that previously showed activity on carboxylic acids including 2-naphthoic acid, 4-ethylbenzoic acid, was engineered through site-directed mutagenesis to endow new capability to convert *p*-coumaric acid to caffeic acid. Using this engineered P450 hydroxylase, 2.8 g/L of caffeic acid was produced through whole-cell biocatalysis [141].

In addition to the direct conversion of *p*-coumaric acid to caffeic acid by C3H as described above, a three-step CoA-dependent pathway has also been identified to transform *p*-coumaric acid to caffeic acid [142–144]. In this novel pathway, *p*-coumaric acid is first converted to *p*-coumaroyl-CoA through the reaction catalyzed by 4CL using acetyl-CoA as a CoA donor [142]. Next, *p*-coumaroyl-CoA is hydroxylated to caffeoyl-CoA by C3H [143]. Lastly, caffeoyl-CoA is hydrolyzed to form caffeic acid by CoA thioesterases [144]. An *E. coli* native hydroxyphenylacetyl-CoA thioesterase having broad substrate spectrum was also shown to act on caffeoyl-CoA to produce caffeic acid [145]. Based on these reaction steps, the CoA-dependent caffeic acid pathway was successfully established in the recombinant *E. coli* by expressing only the *R. glutinis* TAL, *Petroselinum crispus* 4CL and *S. espanaensis* C3H genes [146].

4-Vinylphenol, also known as *p*-hydroxystyrene (*p*-HS), is a versatile petroleum-derived platform chemical that can be used to produce a variety of polymers such as photoresist matrix polymer, resins and coatings. *p*-HS is also used to produce flavoring and fragrance substances in food, beverage and perfume industries [147]. *p*-HS and its derivatives 3,4-dihydroxystyrene and 4-hydroxy-3-methoxystyrene are biosynthesized from *p*-coumaric acid, caffeic acid and ferulic acid, respectively. More specifically, *p*-HS is synthesized from l-TYR via *p*-coumaric acid through the two reactions catalyzed by TAL and phenolic acid decarboxylase (PAD). For 3,4-dihydroxystyrene synthesis, l-TYR is converted to *p*-coumaric acid, caffeic acid and finally to 3,4-dihydroxystyrene through the three reactions catalyzed by TAL, C3H and PAD, respectively. For 4-hydroxy-3-methoxystyrene synthesis, l-TYR is converted to *p*-coumaric acid, caffeic acid, ferulic acid and finally to 4-hydroxy-3-methoxystyrene through the four reactions catalyzed by TAL, C3H, caffeic acid methyltransferase and PAD, respectively. In the shake-flask culture of engineered *E.coli* strain expressing the *S. espanaensis tal* and *sam5*, *Bacillus amyloliquefaciens* PAD gene and *A. thaliana* caffeic acid methyltransferase gene, the titers of *p*-HS, 3,4-dihydroxystyrene and 4-hydroxy-3-methoxystyrene reached 355, 63 and 64 mg/L [148]. A variety of PADs from different microbial sources have been previously reported and it was shown that *p*-coumaric acid is the preferred substrate compared with caffeic acid and ferulic acid [149]. The engineered *E. coli* expressing the *R. glutinis* PAL gene and *Lactobacillus plantarum* PAD gene produced 0.4 g/L of *p*-HS in glucose-fed batch fermentation. This *p*-HS titer was tenfold higher than that obtained with the *E. coli* strain expressing the *Bacillus substilis* PAD gene. In addition, the cytotoxicity of *p*-HS was observed via the decrease in the respiration curve and loss of *p*-coumaric acid decarboxylase activity [150]. Apart from the well-known microbial hosts, an engineered *S. lividans* expressing the *R. sphaeroides* TAL gene and *Streptomyces sviceus* PAD gene was shown to be prominent for *p*-HS production, as it could assimilate phosphoric acid swollen cellulose and convert it to *p*-HS at the yield of 94 mol% without accumulation of the intermediate *p*-coumaric acid [151]. Due to the toxicity of *p*-HS to microbial cells, the solvent-tolerant *P. putida* S12 was also examined as a host to produce *p*-HS. The engineered *P. putida* strain was constructed by overexpressing the TAL and PAD genes and knocking out the *fcs* gene encoding feruloyl-coenzyme A synthetase involved in competing pathway. In a two-phase water/1-decanol fed-batch fermentation, this engineered *P. putida* strain produced 17.6 g/L of *p*-HS [152].

Flavonoids are natural aromatic compounds produced in plants and fungi, and their skeleton is composed of benzopyrano moiety and benzene ring. According to the presence of additional rings and reduction/oxidation on pyrano ring, flavonoids are categorized into the subclasses of flavanols, flavanones and isoflavones. In natural biosynthesis of flavonoids, both l-PHE and l-TYR serve as precursors towards the formation of flavanone chalcones followed by formation of flavanones and flavonoids. At first, *p*-coumaric acid is produced either from l-PHE via CA through the reactions catalyzed by PAL and C4H or from l-TYR catalyzed by TAL as described above. Then, *p*-coumaric acid is converted by 4CL to *p*-coumaroyl-CoA, which is reacted with three molecules of malonyl-CoA by chalcone synthase (CHS) to yield a C6–C3–C6 backbone unit “flavanone chalcone of naringenin”. Subsequently, this chalcone is converted to flavanone by non-enzymatic reaction or chalcone isomerase (CHI), and also serves as a precursor for the biosynthesis of other flavonoids [153].

Biosynthesis of the flavonoids pinocembrin and naringenin has been performed in engineered *E. coli*. On the basis of its broad substrate specificity, the *S. coelicolor* A3(2) 4CL, together with the *Rhodotorula rubra* PAL/TAL, was employed to simultaneously produce cinnamoyl-CoA and *p*-coumaroyl-CoA from l-PHE and l-TYR, respectively. These two CoA intermediates were further converted to the corresponding chalcones of pinocembrin and naringenin by CHS originating from the plant *Glycyrrhiza echinata*. The engineered *E. coli* harboring the above synthetic pathway produced 0.27 and 0.17 µg/L of naringenin and pinocembrin from glucose in shake-flask experiments, with the accumulation of 0.47 and 1.23 mg/L of *p*-coumaric acid and CA, respectively. When 2 mM l-PHE and l-TYR were supplemented to increase precursor supply, 1.2- and 2-fold increase in the titers of pinocembrin and naringenin were observed [154]. When the genes encoding the plant-originated *A. thaliana* C4H, *Petroselinum crispum* 4CL and *Petunia *× *hybrida* CHI and CHS were expressed in engineered *S. cerevisiae* using CA and *p*-coumaric acid as substrates, the titers of pinocembrin and naringenin reached 16.3 and 28.3 mg/L, respectively. In another study, a recombinant *E. coli* was constructed by expressing the PAL, 4CL, CHS and CHI genes for pinocembrin synthesis. This recombinant strain was further engineered to enhance the malonyl-CoA pool by applying the CRISPR interference system. The resulting strain produced 525.8 mg/L of pinocembrin from glucose in a two-stage pH-controlled fed-batch culture [155]. Moreover, microbial production of eriodictyol was also achieved with a titer of 6.5 mg/L when caffeic acid was added [156].

In previous studies, the formation of *R*- and *S*-form of flavonoids was not taken into consideration. However, it was reported that a racemic mixture of flavonoids was synthesized by the engineered *E. coli* with the lack of CHI activity. Also, it was identified that a bottleneck in the production of flavonoid was the limited pool of malonyl-CoA that was derived from acetyl-CoA by acetyl-CoA carboxylase (ACC). Engineered *E. coli* expressing the *Pueraria lobate* CHI and *C. glutamicum* ACC genes produced 1.01 mg/L of naringenin and 0.71 mg/L of pinocembrin in the presence of 2 mM l-TYR and l-PHE, respectively. When the concentrated suspension of engineered cells was supplied with 3 mM l-PHE and l-TYR, the pinocembrin and naringenin titers were increased to 58 and 57 mg/L, respectively [157]. Co-expression of the *Photorhabdus luminescens* ACC gene in the engineered *E. coli* strain expressing the *Petroselinum crispum* 4CL, *Petunia hybrida* CHS and *Medicago sativa* CHI genes, the higher titers of pinocembrin, naringenin and eriodictyol reached 196, 67 and 17 mg/L, respectively. Since ACC is a biotin-dependent carboxylase related to the biotin carboxyl carrier protein, the *birA* gene encoding a biotin-ligase was co-expressed with the ACC gene along with external addition of biotin. The resultant *E. coli* produced 367 mg/L of pinocembrin, 39 mg/L of naringenin and 50 mg/L of eriodictyol. By further overexpressing the *E. coli acs* gene encoding acetyl-CoA synthetase, the highest titers of pinocembrin, naringenin and eriodictyol of 429, 119 and 52 mg/L were achieved, respectively. This was caused by the increase in cellular acetyl-CoA availability through acetate assimilation by the engineered *E. coli* strain. However, overexpression of the genes encoding acetate kinase and phosphate acetyltransferase resulted in slightly lower titers of these flavonoids compared with those obtained with the *acs* overexpression [158]. Modulation of the fluxes in other metabolic pathways such as malonate assimilation pathway and fatty acid pathway, was also shown to enhance production of pinocembrin, naringenin and eriodictyol [159].

Although flavonoids have been produced by engineered microbial systems as mentioned above, there is a drawback associated with the use of economically infeasible phenylpropanoic precursors. To replace these precursors with glucose, the pathway engineering strategies such as selection of appropriate enzyme candidates from different sources with codon-optimization and engineering malonyl-CoA pool, have been applied towards engineering of the four-step l-TYR-derived flavonoid biosynthetic pathway composed of TAL, 4CL, CHS and CHI. By supplementing cerulenin as a fatty acid enzyme inhibitor, the naringenin titer of 84 mg/L was achieved by the engineered *E. coli* strain from glucose, almost threefold higher than that obtained without cerulenin [111]. Computational methods were also employed to aid the design of favorable strains producing higher amounts of desired products. For example, the application of genome-scale metabolic network modeling was reported to increase the production level of naringenin resulting from enhanced malonyl-CoA availability in engineered *E. coli.* Through deletion of the *fumC* (encoding fumarase) and *sucC* (encoding succinyl-CoA synthase) genes as well as overexpression of the genes encoding ACC, phosphoglycerate kinase and pyruvate dehydrogenase, the engineered *E. coli* produced 474 mg/L of naringenin with the supplementation of *p*-coumaric acid [160]. When one or more gene interventions predicted by in silico flux analysis are carried out, an optimally balanced metabolic flux in the cell should be considered to avoid intermediate accumulation. To achieve balanced fluxes, the modular approach with the choice of appropriate gene copy number and promoter strength was also suggested [161].

The aforementioned artificial flavonoid pathway has also been expanded in microbial cell factories to produce various other aromatic natural products such as flavones (chrysin, apigenin, luteolin) and flavonols (kaempferol, quercetin, garbanzol, fisetin). By introduction of the soluble flavone synthase1 (FSI) from parsley into the aforementioned biosynthetic pathway established in *S. cerevisiae* [156], naringenin, pinocembrin and eriodictyol were converted to apigenin, chrysin and luteolin, respectively. By replacing FSI with the snapdragon insoluble flavone synthase II (FSII) (encoded by *AFNS2*) along with co-expression of the *S. cerevisiae* cytochrome P450 reductase (CPR1) gene, the apigenin titer was increased to 46 and 57 μg/L in raffinose- and acetate-containing media, respectively. A higher luteolin titer was achieved by using raffinose rather than acetate as carbon source. Chrysin production was eliminated due to the replacement of FSI with FSII [162]. To further produce methylated apigenin and luteolin, a recombinant *E. coli* strain was constructed by expressing the peppermint *OMTIA* gene (encoding 7-*O*-methyltransferase), parsley *4CL* and *FSI* genes, and the petunia *CHS* and *CHI* genes. The titers of apigenin and the methylated form of apigenin called genkwanin reached 415 and 208 μg/L, respectively, when *p*-coumaric acid was fed in the presence of FSI activity. However, the luteolin titer was rather low and thus the methylated luteolin was not detected in the medium by using caffeic acid as carbon source [163]. By introducing the *Citrus sinensis F3H* (encoding flavanone 3-hydroxylase) and *Citrus unshiu FLS* (encoding flavanol synthase) genes into the engineered *E. coli* strain harboring the flavonoid biosynthetic pathway through expressing the genes encoding PAL/TAL, 4CL, CHS and CHI, 15.1 mg/L of kaempferol and 1.1 mg/L of galangin were achieved from l-TYR and l-PHE, respectively. Also, chrysin and apigenin were produced to concentrations of 9.4 and 13.1 mg/L from l-PHE and l-TYR, respectively, by using the *P. crispum* FSI [164]. Using a fusion protein of the *Catharanthus roseus* 3′,5′-hydroxylase (F3′5′H) with CPR, the titers of kaempferol and quercetin were increased to 140 and 20 μg/L, respectively, compared to those obtained without the chimeric enzyme. However, myricetin production was not detected [165]. Other chimeric enzymes including the *Glycine max* isofalavone synthase (ISFI) and *C. roseus* CPR were also employed in engineered *E. coli* and *S. cerevisiae* for the production of genistein and daidzein from naringenin and liquiritigenin, respectively. The product yield of genistein and daidzein in engineered bacteria reached 10 and 18 mg/g DCW, respectively, 20- and 9-fold higher than those obtained in engineered yeasts [166]. In another study, four engineered *S. cerevisiae* strains were constructed by expressing different combinations of genes encoding the following enzymes, PAL and CPR from *Populus trichocarpa *×* P. deltoides*, C4H, 4CL, CHS, CHI, F3H, F3′H and IFS from *G. max*, and FLS from *Solanum tuberosu.* These engineered yeast strains (named GEN23, KAE34, QUE44 and RESV11) enabled the production of genistein, kaempferol, quercetin and resveratrol from l-PHE [167]. The glucoside forms of isoflavonoids such as genistein 4′-*O*-β-d-glucoside, genistin, genistein 4′,7-*O*-β-d-diglucoside, sissotrin, daidzein 4′,7-*O*-β-d-diglucoside, daidzein 4′-*O*-β-d-glucoside, daidzin, and ononin were also produced from respective isoflavonoid precursors catalyzed by the *Bacillus licheniformis* glycosyltransferase (encoded by *yjiC*) in engineered *E. coli* [168]. For fisetin synthesis, an artificial metabolic pathway was constructed, which was inspired from the quercetin pathway due to the structural similarity between them. Specifically, l-TYR is sequentially converted *p*-coumaric acid, *p*-coumaroyl-CoA, isoliquiritigenin, liquiritigenin, garbanzol, resokaempferol and finally to fisetin through the reactions catalyzed by TAL, 4CL, CHS-CHR, CHI, F3H, FLS and FMO/CPR, respectively. Engineered *E. coli* harboring this synthetic pathway produced 2.1 mg/L of fisetin with supplementation of resokaempferol [169]. Using a similar pathway approach, the engineered *S. cerevisiae* produced 20 mg/L of quercetin and 26 mg/L of kaempferol (Fig. 3) [170].

Microbial production of resveratrol has been achieved in engineered microbial systems based on the natural biosynthesis pathway of stilbenes. By introducing the hybrid poplar 4CL and *Vitis vinifera* resveratrol synthase genes into *S. cerevisiae*, the resveratrol was produced in the range of 100 to 300 nanogram per 200 mL culture [171]. The engineered *E. coli* strain expressing the *A. thaliana* 4CL and *Arachis hypogaea* STS genes produced up to 100 mg/L of resveratrol and 10 mg/L of piceatannol using glycerol as a carbon source with the supplementation of *p*-coumaric acid and caffeic acid, respectively. When glucose was used as carbon source, the resveratrol titer was decreased to 3.8 mg/L, as glucose adversely affected the enzymatic activity and soluble expression of STS [172]. In another study, both engineered *E. coli* and *S. cerevisiae* were constructed for resveratrol production by expressing the plant-originated genes. In the engineered yeast, the *Nicotiana tabacum* 4CL gene was expressed under the control of *GAL10* promoter while the *Vitis vinifera* STS gene was expressed under *GAL1* promoter. The resultant yeast strain produced 6 mg/L of resveratrol. In the engineered *E. coli*, both of the genes were expressed under T7 promoter, which led to a higher resveratrol titer of 16 mg/L [173]. In another study using *S. cerevisiae* for resveratrol production, the engineered *S. cerevisiae* was first constructed by expressing the *H. aurantiacus* TAL, *A. thaliana* 4CL and *V. vinifera* STS genes. Then, the *aro4*^fbr^ (encoding a feedback-insensitive DAHP synthase), *aro7* (encoding CHA mutase) and *ACC1* (encoding a post-translational de-regulated acetyl-CoA carboxylase) genes were further overexpressed to enhance precursors supply of tyrosine and malonyl-CoA. This final strain produced 415.65 and 531.41 mg/L of resveratrol from glucose or ethanol in fed-batch fermentation, respectively [174]. A strategy of the facilitated transfer of cascade intermediates between 4CL and STS was applied by fusing the two enzymes together. For this purpose, the stop codon of *A. thaliana* 4CL gene was replaced with a linker sequence followed by the *V. vinifera* STS gene sequence. When this 4CL-STS fusion protein was expressed in *S. cerevisiae*, 5.25 µg/mL of resveratrol was produced, a 15-fold increase than that obtained by the simple co-expression of these enzymes [175]. On the basis of resveratrol synthesis, various glycoside derivatives of resveratrol, such as resveratrol-4′-*O*-glucoside, resveratrol-3-*O*-glucoside and resveratrol-3-*O*-glucoside, have also been produced by engineered microbes by further expressing the *B. licheniformis* glycosyltransferase with broad substrate specificity against diverse nucleotide diphosphate sugars [176].

### l-TRP pathway derivatives

In comparison to l-PHE and l-TYR pathways, l-TRP pathway has been relatively less studied, and thus has the greater potential to be exploited for biosynthesis of more and novel derivative aromatic compounds. A few l-TRP derivatives that have been produced by microbial systems are listed in Table 4.

**Table 4:** The system metabolic engineering strategies on tryptophan pathway for the microbial production of aromatic compounds

Product	Precursor	Carbon source	Host	Titer (g/L)	Time (h)	Bioprocess strategy	Systems metabolic engineering strategies	References
Anthranilic acid	CHA	Glucose	*E. coli*	14	34	Fed-batch (fermentor, 1 L)	Random mutagenesis in gene *trpD* encoding anthranilate phosphoribosyl transferase; Overexpressing following genes encoding feedback resistant DAHP and transketolase	[180]
Serotonin	l-TRP	Glucose	*E. coli*	0.1543	24	Two-step fermentation (fermentor, 1.5 L)	Introducing gene encoding aromatic amino hydroxylase in one host; Introducing gene encoding tryptophan decarboxylase in tryptophanase knock-out strain	[184]
Indigo	l-TRP	Glucose	*E. coli*	18	~ 72	Fed-batch (fermentor, 14 L)	Introducing gene encoding naphthalene dioxygenase; Common metabolic pathway engineering strategies: knocking-out genes encoding pyruvate kinase I and II, and overexpressing genes feedback resistant DAHP and transketolase	[186]
Indirubin	l-TRP	Tryptophan	*E. coli*	0.2236	48 h	Batch (fermentor, 10 L)	Integrating novel gene encoding flavin-containing monooxygenase (FMO) from *Methylophaga aminisulfidivorans*; Investigating the effect of cysteine and oxygen on indirubin synthesis	[188]
Violacein	l-TRP	Glucose	*C. glutamicum*	5.436	~ 120	Fed-batch (fermentor, 3 L)	Introducing *vio* operon vioABCDE from *Chromobacterium violaceum*; Replacing RBS in vio genes; Overexpressing them under inducible promoter; Optimizing induction time	[195]
Deoxy-violacein	l-TRP	Glycerol	*E. coli*	1.6	200	Fed-batch (fermentor, 0.7 L)	Integrating and overexpressing *vio* operon vioABCE under the control of *araBAD* promoter; Deleting *araBAD* gene; Inducing by l-arabinose	[192]

Anthranilic acid is a metabolite of l-TRP biosynthesis pathway in microorganisms [177], and is also present in the carbazole degradation pathway of *Pseudomonas stutzeri* [178]. Currently, anthranilic acid is chemically synthesized from petroleum-derived *o*-xylene, and is mainly used in the synthesis of dyestuff, perfumes and pharmaceuticals [179]. To enable anthranilic acid accumulation by blocking the l-TRP pathway, a mutant *E. coli* strain having a nonsense mutation in the *trpD* gene (encoding the anthranilate phosphoribosyl transferase component of the anthranilate synthase-phosphoribosyl transferase complex) was generated by random mutagenesis. Overexpression of the genes that encode a feedback-resistant DAHP synthase and transketolase in this mutant *E. coli* resulted in production of 14 g/L of anthranilic acid in fed-batch culture [180]. In another study, an engineered *P. putida* strain was also developed to produce anthranilic acid. In this engineered *P. putida*, the *trpDC* operon encoding anthranilate phosphoribosyltransferase (TrpD) and indole-3-glycerol phosphate synthase (TrpC), and the *pheA* gene encoding chorismate mutase, were knocked out to eliminate competitive pathways. Further overexpression of the *aroG*^D146N^ and *trpE*^S40F^*G* genes encoding the feedback-insensitive versions of DAHP synthase and anthranilate synthase, respectively, in this mutant *P. putida* strain led to production of 1.54 g/L of anthranilic acid from glucose in a tryptophan-limited fed-batch culture [181].

Serotonin (5-hdyroxytryptamine) acts as a neurotransmitter in animals and also plays a role in regulating various physiological functions in plants. Serotonin is synthesized via l-TRP in both animals and plants but through different biosynthetic mechanisms. In animals, l-TRP is converted to serotonin via 5-hydroxytryptophan through the sequential reactions catalyzed by tryptophan 5-hydroxylase (TPH) and aromatic amino acid decarboxylase. Whereas in plants, l-TRP is first converted to tryptamine and then to serotonin by tryptophan decarboxylase (TDC) and tryptamine-5-hydoxylase (T-5H) [182]. This plant serotonin pathway was successfully established in a recombinant *E. coli* overexpressing the *Catharanthus roseus* TDC and rice T-5H genes. The soluble expression of T-5H was optimized by replacing the N-terminal 37 amino acid residues with a glutathione S transferase (GST) tag. Under optimized condition, this engineered *E. coli* strain produced 24 mg/L of serotonin [183]. In a recent study, a stepwise system comprising two recombinant *E. coli* strains was constructed for serotonin production based on the animal serotonin pathway. In the first step, 962 mg/L of 5-hydroxytryptophan was produced from glucose by the recombinant *E. coli* strain expressing the gene encoding a mutated aromatic amino acid hydroxylase (CtAAAH) from *Cupriavidus taiwanensis*. Subsequently, bioconversion of the 5-hydroxytryptophan obtained in the first step led to production of 154.3 mg/L of serotonin by a second recombinant *E. coli* strain expressing the *C. roseus* TDC gene [184].

Indigo is a blue dye compound, which is obtained either by plant extraction or chemical synthesis. In some microorganisms such as *Pseudomonas* species, indigo is found to be produced from either indole or glucose as well as from degradation of particular aromatic compounds. Indigo and its isomer indirubin are synthesized from l-TRP by a three-step bioconversion: first, l-TRP is deaminated to indole by tryptophanase; then, indole is simultaneously oxidized to isatin, 2- and 3-hydroxyindole by monooxygenases or dioxygenases; lastly, spontaneous dimerization of these intermediates results in the formation of indigo and indirubin [185]. A recombinant *E. coli* strain expressing the *P. putida* naphthalene dioxygenase gene was constructed for indigo production. This *E. coli* was further engineered to enhance the l-TRP pathway flux by overexpressing the *aroG*^fbr^ and *tktA* genes encoding a feedback-resistant DAHP synthase and transketolase, respectively, and knocking out the *pykF* and *pykA* genes encoding pyruvate kinase I and II, respectively. The resultant engineered *E. coli* produced 18 g/L of indigo from glucose in fed-batch culture [186]. On the other hand, the monooxygenase-mediated indigo production was also explored. A recombinant *E. coli* strain expressing the *Methylophaga aminisulfidivorans* MP^T^ *fmo* gene encoding a flavin-containing monooxygenases (FMO) produced 920 mg/L of indigo from 2 g/L of l-TRP in a batch culture [187].

Indirubin (also known as couraupitine B) is an indole alkaloid that serves as a drug substance used in the treatment of various diseases such as granulocytic leukemia, cancer and Alzheimer’s disease. Indirubin is currently produced either by plant extraction or chemical synthesis. Production of indirubin has also been achieved in engineered microbial systems. In addition to indigo production, the aforementioned recombinant *E. coli* strain expressing the *fmo* gene also produced 5.0 mg/L of indirubin from l-TRP. When this recombinant *E. coli* was cultured with supplementation of 0.36 g/L of cysteine, a higher indirubin titer of 223.6 mg/L was achieved. It was speculated that the addition of cysteine enhanced synthesis of the indirubin precursor 2-hydroxyindole through affecting the regioselectivity of FMO [188]. Recently, microbial production of indirubin from glucose was accomplished by constructing an *E. coli* strain expressing the *M. aminisulfidivorans fmo* and *E. coli* tryptophanase-encoding *tnaA* genes. This recombinant *E. coli* strain was further engineered by knocking out *trpR* (encoding a regulatory repressor), *pykF* (encoding pyruvate kinase I) and *pykA* (encoding pyruvate kinase II), and overexpressing *aroG*^fbr^ (encoding a feedback-resistant DAHP synthase), *trpE*^fbr^ (encoding a feedback-insensitive anthranilate synthase) and *tktA* (encoding transketolase). The resultant engineered *E. coli* strain successfully produced 56 mg/L of indirubin from glucose in fed-batch culture [189].

Violacein and deoxyviolacein are secondary metabolites found in microorganisms such as *Collimona*, *Duganella*, *Janthinobacterium* and *Pseudoalteromonas*, and are synthesized by condensation of two tryptophan-derived molecules [190]. Violacein and deoxyviolacein are considered as potential drug agents in pharmaceutical industry because of their anti-microbial, anti-tumor and anti-oxidant activities [186, 187]. Violacein is synthesized from l-TRP by the *vioABCDE* operon that encodes VioA, VioB, VioE, VioD and VioC, while deoxyviolacein synthesis requires the same set of enzymes except VioD [191]. A recombinant *E. coli* strain expressing the *Chromobacterium violaceum vioABCE* operon produced 180 mg/L of deoxyviolacein from glucose in shake flask cultures. Through systems-wide engineering of the serine, CHA and l-TRP biosynthesis and the non-oxidative PP pathway, bottlenecks in l-TRP supply were eliminated. As a result, a higher deoxyviolacein titer of 320 mg/L was obtained. Furthermore, co-expression of the *Janthinobacterium lividum vioD* gene in the above *E. coli* strain resulted in exclusive production of 710 mg/L of violacein from glucose in a fed-batch process [191]. In a following study, the *C. violaceum vioABCE* operon was overexpressed in a recombinant *E. coli* using the *araBAD* promoter induced by l-arabinose. In this *E. coli* strain, l-arabinose metabolism was also eliminated to prevent catabolism of the inducer l-arabinose by knocking out the *araBAD* genes. Using the resultant *E. coli* strain, 1.6 g/L of deoxyviolacein was achieved in a glycerol-based fed-batch process [192]. Using another *vioABCDE* cluster from *Duganella* sp. B2, violacein production was investigated in three different bacterial hosts including *E. coli*, *Citrobacter freundii* and *Enterobacter aerogenes*. As a result, the highest violacein titer of 1.68 g/L was achieved by the recombinant *C. freundii* strain expressing the *Duganella* sp. B2 *vioABCDE* operon in shake flask cultures [193]. When this recombinant *C. freundii* strain was cultured in a glycerol fed-batch process with optimized condition, 4.13 g/L of violacein was obtained [194]. In another study, recombinant *C. glutamicum* strains were constructed by overexpressing the *C. violaceum vioABCDE* operon using different expression strategies, including the use of constitutive or inducible promoter, RBS replacement and reorganization of the *vio* operon. The best recombinant *C. glutamicum* strain produced 5.436 g/L of violacein from glucose in an optimized fed-batch process [195].

Another interesting l-TRP derivative aromatic compound that has been recently studied is indole-3-acetic acid, also called auxin. Auxin is one of the phytohormones found in plants and is also synthesized in a range of phytobacteria such as Salmonella. A key enzyme for auxin synthesis called indole-3-pyruvate decarboxylase (IpdC) was identified in Salmonella. Heterologous expresssion of the Salmonella ipdC gene in the plant *Medicago truncatula* led to auxin production [196]. In the future, it is of great interest to explore auxin production in a recombinant microbial host expressing the Salmonella ipdC gene.

## Conclusions

In this paper, we reviewed the current status of production of natural and non-natural aromatic chemicals by metabolically engineered microorganisms. Various metabolic engineering strategies and tools employed in the relevant works were reviewed. The aromatic chemicals that have been produced are classified according to their key precursors including SHK and aromatic amino acids. Some of the chemicals showcased in this paper are still produced at rather low efficiency, and thus need further strain improvement to enhance their production. It is expected that more and more aromatic compounds of industrial interest will be produced by microbial cell factories, either through the innovative rewiring of existing pathways or by identification of new enzymes and pathways. Complex regulatory circuits responsible for limited metabolic flux towards aromatic amino acids formation have been quite well known and thus do no longer present obstacle in increasing fluxes. For many aromatic products, in particular complex natural compounds, their production in engineered microorganisms is often limited by the activities of heterologous enzymes introduced for their production. Identification of better enzymes and also further improvement of these enzymes will play important roles in further enhancing production of various aromatic chemicals. As in the cases for developing strains overproducing other chemicals, systems metabolic engineering that integrates metabolic engineering with systems biology, synthetic biology and evolutionary engineering will offer more streamlined design of strategies for developing microbial strains efficiently producing various aromatic chemicals [5, 197].
